# PhysioZoo: A Novel Open Access Platform for Heart Rate Variability Analysis of Mammalian Electrocardiographic Data

**DOI:** 10.3389/fphys.2018.01390

**Published:** 2018-10-04

**Authors:** Joachim A. Behar, Aviv A. Rosenberg, Ido Weiser-Bitoun, Ori Shemla, Alexandra Alexandrovich, Eugene Konyukhov, Yael Yaniv

**Affiliations:** ^1^Faculty of Biomedical Engineering, Technion-IIT, Haifa, Israel; ^2^Faculty of Computer Science, Technion-IIT, Haifa, Israel

**Keywords:** heart rate variability, electrocardiography, animal models, R-peak detection, power law

## Abstract

**Background:** The time variation between consecutive heartbeats is commonly referred to as heart rate variability (HRV). Loss of complexity in HRV has been documented in several cardiovascular diseases and has been associated with an increase in morbidity and mortality. However, the mechanisms that control HRV are not well understood. Animal experiments are the key to investigating this question. However, to date, there are no standard open source tools for HRV analysis of mammalian electrocardiogram (ECG) data and no centralized public databases for researchers to access.

**Methods:** We created an open source software solution specifically designed for HRV analysis from ECG data of multiple mammals, including humans. We also created a set of public databases of mammalian ECG signals (dog, rabbit and mouse) with manually corrected R-peaks (>170,000 annotations) and signal quality annotations. The platform (software and databases) is called PhysioZoo.

**Results:** PhysioZoo makes it possible to load ECG data and perform very accurate R-peak detection (*F*_1_ > 98%). It also allows the user to manually correct the R-peak locations and annotate low signal quality of the underlying ECG. PhysioZoo implements state of the art HRV measures adapted for different mammals (dogs, rabbits, and mice) and allows easy export of all computed measures together with standard data representation figures. PhysioZoo provides databases and standard ranges for all HRV measures computed on healthy, conscious humans, dogs, rabbits, and mice at rest. Study of these measures across different mammals can provide new insights into the complexity of heart rate dynamics across species.

**Conclusion:** PhysioZoo enables the standardization and reproducibility of HRV analysis in mammalian models through its open source code, freely available software, and open access databases. PhysioZoo will support and enable new investigations in mammalian HRV research. The source code and software are available on www.physiozoo.com.

## Introduction

Over the past few decades, numerous studies have explored the variation of the time interval between heartbeats, also known as HRV. Studies have shown that measures quantifying HRV together with heart rate (HR) can provide useful information on cardiovascular health (Pincus and Goldberger, 1994; Yaniv et al., 2015b). Use cases include prediction of sepsis in neonates (Lake et al., 2002), detection of atrial fibrillation (Oster et al., 2013; Behar et al., 2017), detection of obstructive sleep apnea (Roche et al., 1999), or identifying intrauterine growth restricted fetuses (Henson et al., 1984). Importantly, loss of the so-called “complexity” in the HRV of a patient with cardiovascular disease has been correlated with an increase in both morbidity and mortality. Understanding the mechanisms that contribute to these changes is therefore essential. In recent years interest in HRV analysis has increased due to (i) the existence of large, publicly available biosignal databases [e.g., the Research Resource for Complex Physiologic Signals, or PhysioNet (Goldberger et al., 2000)], or similar private counterparts; (ii) the development of more advanced digital signal processing algorithms for exploiting the physiological content of the beat-to-beat interval time series; and (iii) the availability of affordable, wearable medical devices and implantable telemetry devices from which continuous heart rate time series can be obtained. Despite these encouraging studies, the mechanisms that control HRV are not yet well understood, and the use of HRV analysis has remained limited in medical practice.

With the recent advances in genome manipulation technologies, animals with mutations designed to overexpress or knock out genes implicated in human cardiovascular diseases have become an important focus of biological research (Thireau et al., 2008). HRV is a non-invasive tool that can be used to analyze the electrical heart activity of mutant animals and provide new insights into the pathophysiology and how these conditions may be diagnosed. HRV analysis has also been used to study the role of cardiac mediators and signaling pathways in heart rhythm (Uechi et al., 1998; Witte et al., 2004) and the effect of pharmacological substances on the HRV (Elghozi et al., 2001; Tank et al., 2004). In addition, HRV can be used to characterize functional changes at different levels of integration (Yaniv et al., 2013b, 2014) (i.e., whole heart, sinoatrial tissue, and single pacemaker cells) in disease or aging (Yaniv et al., 2016). Such experiments can only be performed using animal models.

Despite the clear motivation for integrating HRV analysis into animal studies, some drawbacks prevent researchers from doing so: (i) there are no standard publicly available R-peak detector algorithms adapted for use with mammalian electrophysiological data; (ii) the HRV measures and available software implementing them are based on human electrophysiological data analysis, and there is no standard method for adapting them to other mammals; and (iii) there is no standardized annotated database that can be used as a reference when developing new programs for HRV analysis or new HRV indices. These limitations have motivated our creation of the PhysioZoo platform for HRV analysis of mammalian electrophysiological data. This new platform includes a set of standardized databases of electrophysiological data from common, healthy animals, an R-peak detector for accurately estimating the beat-to-beat intervals from electrophysiological data of different mammals, and software implementing the state of the art HRV measures, with parameters adapted to each species. PhysioZoo was implemented in MATLAB (The MathWorks, Inc., Natick, MA, United States). A standalone compiled version of the software is also available for users without a MATLAB license.

The platform introduced in this paper thus represents the first step toward tackling the aforesaid limitations. The PhysioZoo platform enables the standardization and reproducibility of HRV analysis for human, dog, rabbit, and mouse electrocardiographic (ECG) data through its open source code, freely available software, and open access ECG databases. PhysioZoo will support and enable new developments in mammalian HRV research. We made available the source code, software and documentation on physiozoo.com and the databases on physionet.org.

## Materials and Methods

### Databases and Annotations

Electrocardiographic data from dogs (Billman, 2013), rabbits (Brunner et al., 2008; Odening et al., 2012), and mice (Yaniv et al., 2016) were obtained. All animal data used in the present paper were obtained from published studies for which the respective animal protocols and experimental procedures were approved by the original research committee (Brunner et al., 2008; Odening et al., 2012; Billman, 2013; Yaniv and Maltsev, 2014; Yaniv et al., 2016). Human ECG data were obtained from the public MIT-BIH Normal Sinus Rhythm (MIT-NSR) database (Goldberger et al., 2000). Human data consist of 18 long-term ECG recordings recorded at 128 Hz from individuals who had no significant arrhythmias. Dog data were recorded at 500 Hz, and body surface electrodes were placed on either side of the animal’s chest and secured with surgical tape. Rabbit data were recorded by means of subcutaneous ECG recording using the Ponemah platform (DSI, MN, United States) at 1 kHz. ECG raw data were exported to text files from the Ponemah software using the maximal precision (four decimal places). All rabbits were female and free moving in a cage. Mouse data were recorded using Telemetry sensors (ETA-F20 or HDX-11, Data Sciences International, Saint Paul, MN, United States) with a sampling rate of 1000 Hz. All the mammals were conscious, and no drugs were administered prior to the recording. When multiple channels of ECG were available, only the first channel was considered for analysis and stored in our databases.

**FIGURE 1:**
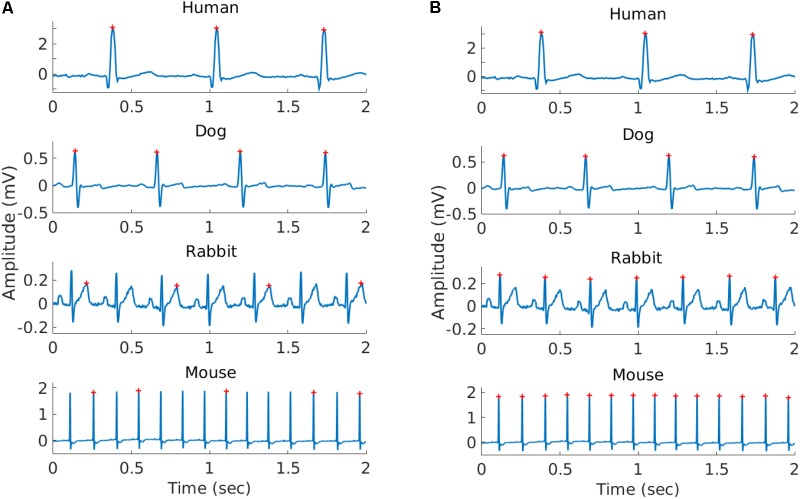
Representative example of the R-peaks detected using gqrs (left) and rqrs (right) on the same 2 s segment. **(A)** R-peaks detected by gqrs (state-of-the-art human R-peak detector). This figure illustrates the need for mammal-specific R-peak detectors to ensure accuracy. **(B)** R-peaks detected by rqrs (i.e., adapted for working with mammalian data).

We performed peak detection to identify the R-peak locations (Behar et al., 2014b) in the animal databases. For the human database, the reference R-peaks available on PhysioNet were used (Goldberger et al., 2000). Because no state of the art R-peak detector has ever been designed and evaluated for data from animals (whose beating rates differ from that of humans; see **Figure 1**), we manually corrected the peak locations. For that purpose, a single trained annotator reviewed all the recordings and corrected the inaccurate annotations (false positive and false negative). In addition, segments were marked as bad quality when no R-peak could be visually identified by the annotator for at least three consecutive peaks. Annotations within these low quality segments were removed. Thus, for each record in our database, we obtained the reference (i.e., human corrected) R-peak annotations and the signal quality annotations (see **Table 1** and **Supplementary Tables S4–S6** for a summary of the databases). We used these reference annotations to evaluate the mammal-specific R-peak detector implemented in the PhysioZoo software as well as to provide standard ranges of the HRV measures.

**Table 1:** Summary of the PhysioZoo database content.

	Human	Dog	Rabbit	Mouse
Number of records	18	17	20	8
Number of mammals	18	17	4	8
Average length (hr:min:sec)	24:18:18	00:05:31	00:10:34	00:29:44
Min length (hr:min:sec)	23:08:00	00:04:09	00:04:53	00:13:53
Max length (hr:min:sec)	25:57:20	00:06:48	00:26:00	00:39:38
Total length (hr:min:sec)	437:29:36	01:33:55	03:31:13	03:28:07
Total R-peak annotations	1,806,792	10,871	50,452	109,865
Bad quality, gross (%)	–	2.7%	1.0%	0.4%

*Data for humans (Goldberger et al., 2000), dogs (Billman, 2013), rabbits (Brunner et al., 2008; Odening et al., 2012), and mice (Yaniv et al., 2016).*

All mammalian ECG data and the manually corrected R-peak reference annotations were contributed to physionet.org (Goldberger et al., 2000).

### Peak Detection

#### R-Peak Detection Algorithm

To calculate HRV measures from ECG signals, we must first detect R-peaks accurately. Numerous algorithms for finding QRS complexes in ECG signals exist and their performance has been studied (Pan and Tompkins, 1985; Llamedo and Martínez, 2014; Johnson et al., 2015). The *gqrs* detector, part of the PhysioNet WFDB Toolkit (Goldberger et al., 2000), has been shown to perform very accurately on several ECG databases (Llamedo and Martínez, 2014). In addition, *gqrs* can be configured to work for animal ECG recordings by changing its configuration parameters.

The *gqrs* algorithm detects the beginning of the entire QRS complex (Q onset) and not the R-peak itself. It is therefore not the best option for HRV calculation, because beat-to-beat analysis of the R-peak provides a more stable fiducial point than the QRS onset. A custom detector, *rqrs*, was created, with an additional step: for each QRS complex detected by *gqrs*, it searches for R-peaks in a small window around the time that the Q onset is detected. In order to ensure that this step works for ECG signals of different polarities, both the minimal and maximal extremal points are searched within the window. The median of the absolute differences between the signal value at the Q-onset (as found by *gqrs*) and the signal amplitude at both extrema are evaluated. If the median difference is larger for the maximal extrema points, the maximal points are taken as R-peaks; otherwise, the minimal points are taken as R-peaks. The duration of the window the extrema are searched in was chosen to be 80% of the average duration of one QRS complex, thus making it species dependent. To ensure that *rqrs* provides robust R-peak detection, at least as accurate as *gqrs* for humans, the algorithm was tested on the updated 2014 PhysioNet Challenge training set of ECG recordings (Silva et al., 2015; see **Supplementary Table S1**).

In order to adapt the R-peak detection algorithm to other mammalian ECG data, some parameter modifications were required. The parameters for humans were taken from the default PhysioNet configuration file. For dog, rabbit, and mouse data, we used the physiological measurements reported in other studies (Hanton and Rabemampianina, 2006; Lord et al., 2010; Sysa-Shah et al., 2015; Chapel et al., 2017; Cintra et al., 2017) and in the Research Animal Resources Center database (University of Wisconsin-Madison, 2018) in order to set the configuration parameters and evaluate the newly configured R-peak detector on our databases.

#### Statistics

To assess the R-peak detection accuracy, the following standard statistical measures were used:

• Sensitivity, *Se*. This is the fraction of correctly detected events (R-peaks),
  $$\mathrm{Se}=\frac{\mathrm{TP}}{\mathrm{TP}+\mathrm{FN}},$$

where *TP* (True positive) is the number of detections that have a matching reference annotation and *FN* (False negative) is the number of reference annotations that were not matched with a detection (missed events).

• Positive Predictive Value, *PPV*. This is the fraction of detections that were actual events (R-peaks):
  $$\mathrm{PPV}=\frac{\mathrm{TP}}{\mathrm{TP}+\mathrm{FP}},$$

where *FP* (false positive) is the number of detections that do not have a matching reference annotation (incorrect detections).

• The overall detection accuracy measure, *F*_1_ (Sasaki and Fellow, 2007), is defined as:
  $$F_{1}=2\frac{\mathrm{PPV Se}}{\mathrm{PPV}+\mathrm{Se}}$$

This statistic was suggested for assessing the performance of R-peak detection algorithms (Behar et al., 2014a) due to its ability to combine multiple fractional measures by using a harmonic mean between the *Se* and *PPV*.

To assess an R-peak detector’s performance, the detected beat locations were compared to the reference manual annotations for that record. For humans, the detection locations were compared to the reference annotations using a 150 ms tolerance window around each detection as standardized by ANSI/AAMI (1998). This tolerance window needs to be mammal dependent to account for the variable HR range across mammals. For each mammal, we defined the tolerance window as 150 ms times the ratio between the human to mammal mean HR. This led to tolerance window values of 90 ms, 40 ms and 17 ms for dogs, rabbits, and mice, respectively. These tolerance windows were used to compute the *Se*, *PPV*, and *F*_1_ statistics for assessing the R-peak detector accuracy for the different mammals. Of note, this tolerance window is relatively large with respect to the RR interval of the corresponding mammal. For humans, for example, a 150 ms window around a reference annotation means that at a typical HR of 60 bpm the tolerance window will be 30% of the RR interval. Thus, practically speaking, an R-peak detector evaluates whether any point on the QRS complex was identified by the algorithm and not the R-peak specifically.

### Heart Rate Variability Measures

#### Prefiltering and Standard HRV Measures

Numerous methods exist to quantify HRV by looking at the beat-to-beat interval length variations; see Malik et al. (1996) and Yaniv et al. (2013a) for reviews. The basic interval lengths can be obtained by measuring the time differences between consecutive R-peaks (RR intervals). However, by definition, only beats resulting from normal sinus node depolarizations (i.e., not arrhythmic, paced, ventricular, etc.) should be used for HRV measures calculations. The intervals between such normal beats are referred to as NN intervals. In order to obtain the NN intervals, RR intervals are found using an ECG R-peak detection algorithm, and then a preprocessing step (either manual or automatic) is performed to filter out suspected ectopic beats, missed beats, and artifacts. We implemented in PhysioZoo three methods for prefiltering the RR-interval: range-based filtering (Mietus and Goldberger, 2017), moving average filtering (Mietus and Goldberger, 2017), and quotient filtering (Piskorski and Guzik, 2005). An example of noise leading to inaccurate R-peak detection is given in **Supplementary Figure S2**. The importance of the prefiltering step for managing these inaccurate detections is illustrated in **Supplementary Figure S3**. **Supplementary Figure S4** shows a comparison of the combined range and moving average filters to the corresponding prefiltering step used in the PhysioNet HRV Toolkit (Mietus and Goldberger, 2017).

A number of HRV measures have been developed over the past three decades. They are traditionally divided into three categories: time domain, frequency domain, and non-linear (Malik et al., 1996). We implemented and included in PhysioZoo the classic HRV measures standardized in 1996 (Malik et al., 1996) and included other measures such as detrended fluctuation analysis (Peng et al., 1995), sample entropy (Richman and Moorman, 2000) and the recently introduced fragmentation HRV measures (Costa et al., 2017).

#### Adaptation to Other Mammals

Because the HR range and dynamics can vary significantly between mammals, some HRV measures need to be adapted. **Figure 1** illustrates the important differences in the HR across mammals and thus the need to adapt some HRV measure parameters. **Table 3** summarizes the parameters selected for each species.

##### Prefiltering

The default parameters for the moving average filter and quotient filter were kept the same for all mammals and with the original parameters used for human RR interval preprocessing (Piskorski and Guzik, 2005; Mietus and Goldberger, 2017). For these two filters, the interface offers three filtering levels: weak, moderate, and strong. For the range filter, because the *RR* is different across mammals, we used the *RR*_min_ and *RR*_max_ defined in **Table 2** for each mammal. Of note, the moving average filter was implemented using zero phase filtering in order to avoid pruning a number of datapoints at the borders and corresponding to the window length. Note that the PhysioNet HRV toolkit prefiltering step does not use a zero phase filter and prunes a non-negligible number of datapoints at the borders (see **Supplementary Figure S4**). This is particularly adverse when using small analysis windows such as the standard 5 min window.

**Table 2:** R-peak detection parameters used to configure rqrs for the different mammalian ECG data.

Parameter	Description	Values				Units
		Human	Dog	Rabbit	Mouse	
HR_m_	Typical heart rate	75	109.5	264	608	[beats/min]
QS_m_	Typical QRS duration	0.07	0.04	0.02	0.00718	[sec]
QT	Typical QT interval duration	0.35	0.19	0.12	0.03	[sec]
RR_min_	Minimum RR interval (“refractory period”)	0.28	0.25	0.14	0.05	[sec]
RR_max_	Maximum RR interval	2.4	1.2	0.58	0.24	[sec]
QRS*_a_*	Typical QRS peak-to-peak amplitude	750	1120	294	1090	[μV]
QRS_amin_	Minimum QRS peak-to-peak amplitude	130	100	114	370	[μV]

*More details about the choice of these parameters are provided in Section “Adaptation of the Parameters for Other Mammals” in **Supplementary Material**.*

##### Time domain measures

The percent of NN interval differences greater than xx milliseconds (denoted pNNxx measure, **Table 3**) uses a fixed criterion for variability, based on a threshold that was found to work well for human ECG data (xx = 50 ms) to quantify vagal activity. This threshold needs to be adapted for different mammals. Assuming that the respiratory rate frequency is characteristic of the vagal activity contribution to HRV, we took the ratio between the average breathing cycle length of the corresponding mammal divided by the average breathing cycle length of humans (rounded value). The respiratory rate range for humans is 12–18 breaths per minute (brpm) (Ganong, 2009), 20–40 brpm for dogs, 30–60 brpm for rabbits, and 60–220 brpm for mice (University of Wisconsin-Madison, 2018). This led to values of 25, 17, and 5 ms for dogs, rabbits, and mice, respectively (see **Table 3**).

**Table 3:** Adaptation of HRV parameters in different mammals.

	Human (Task force)	Human	Dog	Rabbit	Mouse
pNNxx threshold (ms)	50	50	25	17	5
VLF band (Hz)	0.003–0.04	0.0033–0.046	0.0033–0.067	0.0033–0.088	0.0056–0.152
LF band (Hz)	0.04–0.15	0.046–0.158	0.067–0.235	0.088–0.341	0.152–1.240
HF band (Hz)	0.15–0.4	0.158–0.588	0.235–0.877	0.341–1.155	1.240–5
Window size (min)	5	5	5	5	3

*HR, heart rate, LF, low frequency; HF, high frequency; PSD, power spectral density. The “Human (Task force)” column refers to parameters established by the Task Force (Malik et al., 1996). The “Human” column corresponds to the bands we found for humans. “Window size” refers to the typical window size used for PSD analysis. The frequency bands for the different mammals are based on Malik et al. (1996); Thireau et al. (2008), Behar et al. (2018), and the window size for the mouse is based on Thireau et al. (2008).*

##### Frequency domain measures

In healthy humans, the frequency of the PSD performed on a 5-min long RR time series is traditionally divided into three main bands (Malik et al., 1996): the very low frequency (VLF) band, the low frequency (LF) band, and the high frequency (HF) band.

A number of methods exist for spectral estimation. These methods can be broadly categorized as parametric and non-parametric (Porat, 1996). Parametric methods rely on a predetermined model of the process producing the samples, usually an autoregressive (AR) moving average model. Non-parametric methods [typically the Welch (Welch, 1967) or Lomb methods (Lomb, 1976)] compute the spectrum directly usually using Fourier-based analysis of the data itself. In particular, many cardiologists prefer to use the AR model (Carvalho et al., 2003) because it is easier to visually identify the characteristic LF and HF peaks with this “smoother” representation versus the non-parametric methods. While the Lomb method eliminates the need for resampling and thus prevents the artifacts associated with it (Moody, 1993), the risk of aliasing (see **Supplementary Figure S5**) is higher with this method because the maximal frequency we can resolve without aliasing greatly depends on the average HR in the recording. For this reason, we only enable the AR and Welch methods for PSD analysis within the PhysioZoo user interface. See section “Power Spectral Estimation” **Supplementary Material** for more details on the PSD estimation methods.

**FIGURE 2:**
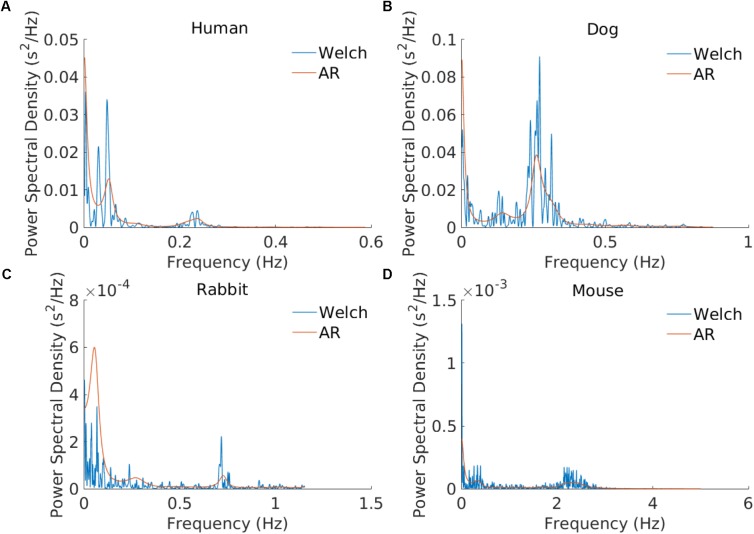
Example of power spectral estimation using an AR model and the Welch method. The three modes corresponding to the VLF, LF, and HF bands are illustrated. AR coefficients: *n* = 20 for the **(A)** human, **(B)** dog, and **(C)** rabbit examples and *n* = 30 for **(D)** the mouse example.

The frequency bands need to be redefined for each mammal because sympathetic and parasympathetic activity manifest at different frequencies for different mammals. **Figure 2** shows an example of PSD obtained using the AR and Welch methods for different mammals and how the frequency bands are to be adapted. For example, the typical peak in the HF band is characteristic of vagal stimulation (Akselrod et al., 1981). On the human database this peak is located at 0.3 Hz, whereas for the mouse database it is located at 1.77 Hz. In order to define mammal-specific bands, we suggested in a recent work (Behar et al., 2018) a Gaussian Mixture model (GMM) to learn these bands with a data-driven approach directly from mammalian ECG databases. The GMM was fitted to the distribution of prominent frequency peaks found in the PSD of the NN time series. We fitted three Gaussians corresponding to the three traditional frequency bands and defined the band’s boundary as the crossing point between consecutive Gaussians. For the mouse data we take a larger upper bound of the HF band at 5 Hz in order to account for the experimental measurements obtained by others (Thireau et al., 2008). For the AR approach to PSD estimation, the order of the AR model must be defined. Boardman et al. (2002) tested different criteria for automatically defining the AR order based on the data and sampling frequency. However, they concluded that a fixed model order should be used because the automated criteria tend to underestimate the AR order. The authors found that an order in the range 16–22 worked well across their human dataset. Analysis of our data showed that an order of 20 was suitable for humans, dogs, and rabbits, and an order of 30 for mice. We set these as the default orders for the respective mammals. An example is provided in **Figure 2**.

**FIGURE 3:**
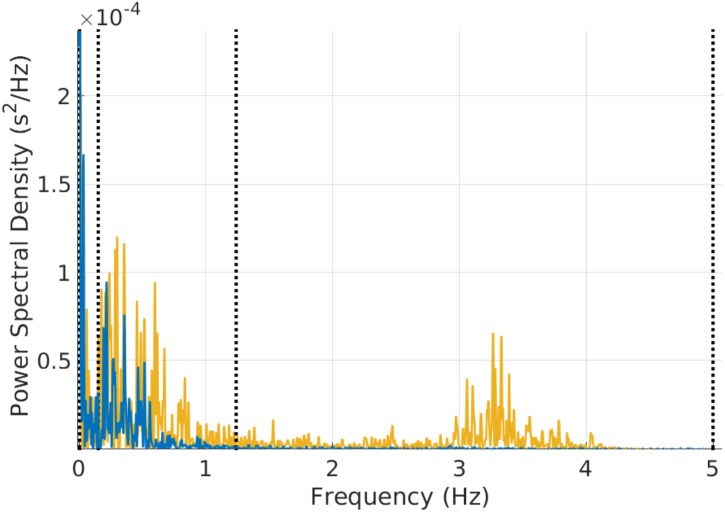
Example of power spectral estimation of a 3 min mouse RR time series before (yellow) and after (blue) atropine injection. The figure shows that the characteristic vagal peak in the mouse-specific HF band is eliminated with atropine. Some of the power in the LF band is also reduced with atropine.

For the AR and Welch PSD estimation methods, a cubic spline was fitted to the NN time series and resampled at a frequency 2.25 times greater than the upper bound of the HF band (thus the resampling rate is relative to the mammal type) in order to comply with the Nyquist criterion. The window size for power spectral analysis is also important and might differ from one mammal to another. We found clear data only for mice; this data, from Thireau et al. (2008), suggests that a window of 3 min is appropriate. For dogs and rabbits, in the absence of further experimental insights, we kept the window size to 5 min as in humans.

##### Non-linear measures

The fractal methods require no adaptation (see section “Detrended Fluctuation Analysis” **Supplementary Material**). However, when using a PSD based estimate of the β coefficient (slope of the linear interpolation of the spectrum in a log-log scale for frequencies below the upper bound of the VLF band), the appropriate VLF frequency band must be used for the corresponding mammal. Entropy methods do not require adaptation for different mammals.

#### Validation

In order to validate the HRV time based measures, we ran the PhysioZoo code on the MIT NSR database (Goldberger et al., 2000) and compared its output to the measures generated by the PhysioNet HRV source codes (HRV toolkit^1^, DFA^2^, and MSE^3^), which are reference source codes for HRV analysis in humans.

### Power and Allometric Laws

To illustrate the type of scientific applications enabled by PhysioZoo, we searched for power and allometric laws between the HRV measures and the typical heart rate (HR_m_) or the typical body mass (BM_m_) of the different mammals included in this study. To search for power laws between the mean HRV measures and HR_m_/BM_m_, we used a double-logarithmic analysis of the mean HRV measures for each mammal type against HR_m_/BM_m_.

## Results

This section presents the results of the performance of the adapted R-peak detector for each mammal type, the PhysioZoo software interface, the range of HRV measures obtained for each mammal type using PhysioZoo to process the data, and an example of use of the program to find the complexity relationship across mammals.

### R-Peak Detector

To validate the ability of *rqrs* to perform at least as well as the standard *gqrs* R-peak detector on human ECG data, the algorithm was tested on the updated 2014 PhysioNet Challenge training set (Silva et al., 2015). The results are presented in **Supplementary Table S1** and show that *rqrs* had *F*_1_ = 93.1% against *F*_1_ = 92.6% for *gqrs*. It is important to note that the input amplitude of the ECG data to *rqrs* must be in millivolts. **Figure 1** shows an example of R-peak detection when using a standard human peak detector (**Figure 1A**) and when using the *rqrs* detector adapted for handling the ECG data of different mammals (**Figure 1B**).

The rqrs R-peak detector was adapted for each mammal (see section “Materials and Methods”) and evaluated on our databases with reference annotations.

**Table 5** provides the performance statistics of the detector for each mammal. The adapted detector very accurately detected the R-peak locations for all mammals, as indicated by the mean and gross statistics (*F*_1_ > 98%). The gross statistics are derived from the sums of all TP, FP, and FN over a mammalian dataset, and mean statistics are the means of the statistics (*Se*, *PPV*, and *F*_1_) calculated individually for each record of a mammalian dataset.

### HRV Algorithms

Standard HRV measures were implemented. **Table 4** summarizes the HRV measures implemented in the PhysioZoo software for the different mammals (dogs, rabbits, and mice). See the supplement for further details and the mathematical background of the HRV measures. All HRV parameters can be manually changed by the user by editing a single configuration file (stored in YAML format). This configuration file may also be uploaded as a supplement to a publication in order to support the reproducibility of the results, as it contains all the parameters required to reproduce the analysis.

**Table 4:** Summary of heart rate variability measures available in the PhysioZoo software.

Measures	Units	Definition
**Time domain**		
AVNN	[ms]	Average NN interval duration
SDNN	[ms]	Standard deviation of NN interval duration
RMSSD	[ms]	The square root of the mean of the sum of the squares of differences between adjacent NN intervals
pNNxx	[%]	Percent of NN interval differences greater than xx milliseconds
SEM	[ms]	Standard error of the mean NN interval
PIP	[%]	Percentage of inflection points in the NN interval time series
IALS	[n.u]	Inverse average length of the acceleration/deceleration segments
PSS	[%]	Percentage of short segments
PAS	[%]	The percentage of NN intervals in alternation segments
**Frequency**		
Total power	[ms^2^]	Total power
VLF	[ms^2^]	Power in the very low frequency band
LF	[ms^2^]	Power in the low frequency band
HF	[ms^2^]	Power in the high frequency band
VLF norm^∗^	[%]	Low frequency power in normalized units *LF/(Total power)* × 100
LF norm	[%]	Low frequency power in normalized units *LF/(Total power – VLF)* × 100
HF norm	[%]	High frequency power in normalized units *HF/(Total power – VLF)* × 100
LF/HF	[n.u]	Low frequency band to high frequency band power ratio (LF/HF)
LF peak	[Hz]	Peak frequency in the low frequency band
HF peak	[Hz]	Peak frequency in the high frequency band
β	[n.u]	Slope of the linear interpolation of the spectrum in a log-log scale for frequencies below the upper bound of the VLF band
**Non-linear**		
SampEn	[n.u]	Sample entropy Richman and Moorman, 2000
SD1	[ms]	NN interval standard deviation along the perpendicular to the line-of-identity
SD2	[ms]	NN interval standard deviation along the line-of-identity
α_1_	[n.u]	DFA low-scale slope Peng et al., 1995
α_2_	[n.u]	DFA high-scale slope

*VLF, very low frequency; LF, low frequency; HF, high frequency; DFA, detrended fluctuation analysis; NN, interval between normal beats. ^∗^Note that the normalization factor for the VLF norm measure (Total power) is different from the LF norm and HF norm ones (Total power-VLF). This is because the LF norm and HF norm measures were defined as such in the HRV standard. In the software we provide the option to change the normalization method to make it the same for all three frequency measures, i.e., divided by the total power.*

The PhysioZoo HRV source code was compared against the HRV source code available from PhysioNet. **Supplementary Table S3** demonstrates the very low NSMSE obtained for all the measures when comparing between the two toolboxes on the MIT NSR databases. The residual differences may be due to errors that result from the difference in rounding between code in MATLAB (PhysioZoo) and C (PhysioNet). However, the errors are minor and will not affect HRV analysis.

### User Interface

After the ECG data is loaded by the user, R-peak detection can be performed, the signal quality can be annotated, and HRV analysis can be performed.

**FIGURE 4:**
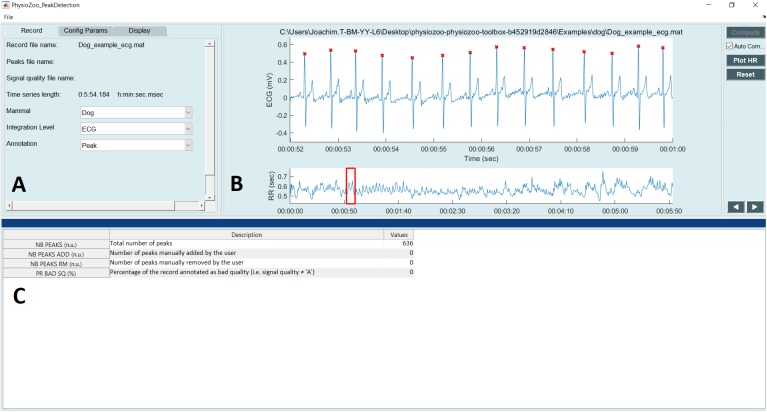
PhysioZoo R-peak detection user interface. **(A)** Record panel. The mammal type dropdown menu is used to load the mammal-specific R-peak detector parameters. Detected R-peaks can be manually corrected using the “Manual annotations” option. Signal quality annotations can be performed. **(B)** Display of the ECG signal (in mV) with the detected R-peaks (red crosses) and RR interval time series. The boxes on the ECG signal highlight a segment labeled as bad quality by the annotator. **(C)** Statistics on R-peak detection and quality annotations.

**Figure 4** shows the interface used for R-peak detection, manual peak correction, and signal quality annotations. Using this interface, ECG data can be loaded (File 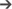 Open data file) and R-peak detection can be performed by clicking the compute button with parameters relative to the mammal type. The interface also allows manual correction of the peak annotations and manual annotation of the signal quality of the underlying ECG. Signal quality provides contextual information on the reliability of the analyzed time series. Different levels of signal quality can be considered in accordance with the analysis goal (Clifford et al., 2012; Behar et al., 2013). Within the context of HRV analysis, we recommend three levels of signal quality, as follows: “A” indicates a very good quality ECG where the R-peaks and morphology (P-wave, T-wave, etc.) can be clearly identified; “B” indicates recordings where the R-peaks can be clearly identified but the quality is too low to allow morphological analysis; and “C” indicates a poor quality ECG where neither R-peak detection nor morphological analysis is possible.

**FIGURE 5:**
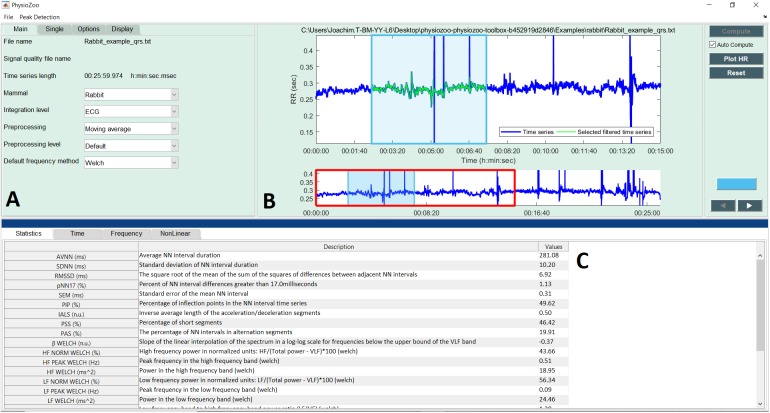
PhysioZoo HRV user interface. **(A)** “Main” tab for choosing the mammal type, RR prefiltering method. The “Analysis” tab can be used for performing multiple window analysis (i.e., batch processing of the time series). The “Options” tab can be used to change all key parameters of the HRV measures. These “Options” are updated automatically in accordance with the chosen mammal; they can also be modified manually. **(B)** Graph displaying the RR or HR time series. In blue the raw time series and in green the filtered time series. **(C)** Panels presenting the HRV measures. Under the tab “Time,” “Frequency,” and “Non-linear,” the HRV measures relative to each of these categories are repeated and the standard figures relative to each category are plotted (e.g., Poincare plots, Power spectrum). More details are available in the software documentation.

**Figure 5** shows the interface for HRV analysis. The PhysioZoo software implements state of the art HRV measures (see section “Materials and Methods”). HRV measure parameters were adapted for different mammals (dog, rabbit, and mouse) and can easily be defined by the user for any other species. All computed measures can be exported together with standard data representation figures. The PhysioZoo software handles data in.txt, .mat (The MathWorks, Inc., Natick, MA, United States) and WFDB (Goldberger et al., 2000) formats. In addition, the PhysioZoo interface enables HRV analysis to be performed on multiple segments, thus allowing changes in HRV measures to be tracked over time for a given record. To load a record, the user should click File 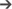 Open data file and select an annotation file for analysis. The HRV measures will be computed automatically. To perform batch processing, the user should click the “Single” menu and select the window size and section of the recording to be analyzed. More information on the user interface and its functionalities is available on physiozoo.com.

### Standard Ranges for HRV Measures

**Table 6** summarizes the median and interquartile interval for all the HRV measures implemented in the PhysioZoo software for all mammals. This provides a standard reference HRV range obtained from conscious and healthy mammals at rest.

### Studying HRV Across Species Using Sample Entropy

**FIGURE 6:**
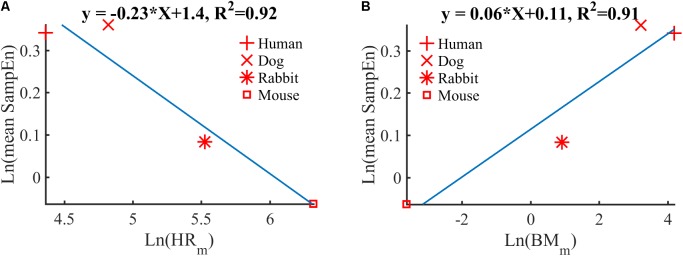
Double-logarithmic plot of: **(A)** the mean SampEn vs. typical heart rate (HR_m_) and **(B)** the mean SampEn vs. body mass (BM_m_).

Using the mean sample entropy (SampEn) measures computed in **Table 6** for each mammal, we found a power law relationship between the mean SampEn and HR_m_ (*R*^2^ = 0.92, **Figure 6A**) and an allometric law between SampEn and BM_m_ (*R*^2^ = 0.91, **Figure 6B**) of the different mammals included in our study. The results suggest that the complexity (represented by sample entropy) of the heart rate increases with HR_m_ and decreases with BM_m_. This in turn suggests that the complexity of the heart rate decreases in smaller mammals.

## Discussion

### PhysioZoo Contributions

Our first contribution is the creation of standardized databases of mammalian electrophysiological data for dogs, rabbits, and mice with reference R-peak annotations and signal quality. These data were obtained for conscious and healthy mammals at rest and contain over 170,000 reference (i.e., manually corrected) R-peak annotations (**Table 1**). A very small fraction of low quality data was included (between 0.4 and 2.7% of the overall data; **Table 1**). These databases can be used as references for future research in mammalian ECG analysis.

Our second contribution is the adaptation of an open source human R-peak detector for other mammals and adaptation of HRV measures to other mammals. Using experimental ranges on the RR interval length and ECG morphology for the different mammals, we configured the *rqrs* peak detector and evaluated its performance on our databases by comparing the output of *rqrs* to the manually annotated R-peak references. The *rqrs* R-peak detector demonstrated high accuracy (*F*_1_ > 98%) for all mammals (**Table 5**). We adapted different HRV parameters to other mammals (see section “Materials and Methods”). For example, we adapted the pNNxx measure to dogs and rabbits. We could not find any in-depth study reporting an optimal threshold value of the pNNxx measure for these species. For mice, Thireau et al., 2008 found the optimal threshold to be 6 ms, which is close to our approximation (i.e., xx = 5 ms, **Table 3**). Similarly, the frequency-based measures were adapted to the mammal type (**Table 3**).

**Table 5:** Mean and gross R-Peak detection, rqrs, performance for human, dog, rabbit, and mouse ECG data.

	Mean (%)			Gross (%)		
	Se	PPV	*F*_1_	Se	PPV	*F*_1_
Humans	96.47	99.87	98.00	96.24	99.87	98.02
Dogs	99.20	98.53	98.84	99.03	98.35	98.69
Rabbits	99.74	99.81	99.77	99.75	99.80	99.77
Mice	98.92	99.87	99.38	98.96	99.88	99.42

**Table 6:** Median (med) and interquartile range (Q1–Q3) of the HRV measures for the mammal databases included in PhysioZoo and from the PhysioNet normal sinus rhythm database for humans.

	HUMAN^∗^ (*p* = 4044, *n* = 16) MED (Q1–Q3)	DOG (*p* = 17, *n* = 17) MED (Q1–Q3)	RABBIT (*p* = 33, *n* = 4) MED (Q1–Q3)	MOUSE (*p* = 64, *n* = 8) MED (Q1–Q3)
AVNN (ms)	786.53	482.63	264.95	108.46
	(692.32–877.22)	(455.02–562.03)	(223.29–281.06)	(101.86–130.70)
SDNN (ms)	53.60	69.20	9.48	10.39
	(40.57–70.85)	(50.80–92.07)	(5.92–12.55)	(5.18–13.72)
RMSSD (ms)	31.28	53.92	4.24	4.55
	(23.73–43.12)	(41.24–89.84)	(2.71–5.45)	(2.61–6.45)
pNNxx (%)	33.06	52.88	0.38	16.46
	(19.87–48.69)	(38.82–69.59)	(0.00–0.95)	(3.06–28.53)
SEM (ms)	2.75	3.10	0.29	0.25
	(2.05–3.76)	(1.98–4.00)	(0.16–0.39)	(0.12–0.34)
PIP (%)	40.60	47.05	41.69	43.09
	(34.48–46.50)	(40.39–53.85)	(39.99–49.68)	(35.47–48.22)
IALS (n.u.)	0.41	0.47	0.42	0.43
	(0.35–0.47)	(0.41–0.54)	(0.40–0.50)	(0.36–0.48)
PSS (%)	37.15	47.08	37.15	38.74
	(26.75–51.35)	(39.31–58.78)	(31.19–46.75)	(27.84–49.69)
PAS (%)	5.81	12.28	15.07	9.32
	(2.40–10.81)	(4.33–19.73)	(10.30–19.82)	(7.13–13.66)
Total power (ms^2^)	2.37e3	4.80e3	56.19	94.57
	(1.32e3–4.21e3)	(2.36e3–7.32e3)	(25.77–120.36)	(22.11–165.88)
VLF (ms^2^)	1.17e3	1.49e3	25.92	47.67
	(597.46–2.26e3)	(980.73–1.88e3)	(15.75–81.78)	(12.66–79.56)
LF (ms^2^)	534.84	707.40	10.48	15.98
	(274.57–988.91)	(397.66–1.94e3)	(5.01–22.61)	(5.07–48.71)
HF (ms^2^)	217.90	1.83e3	4.81	6.98
	(110.16–461.64)	(769.72–4.10e3)	(3.00–9.35)	(2.05–14.49)
VLF norm (n.u.)	54.01	28.64	60.27	49.28
	(41.69–65.41)	(22.63–52.18)	(55.93–69.21)	(39.42–68.06)
LF norm (n.u.)	70.09	32.71	62.69	73.80
	(55.47–81.30)	(24.84–47.40)	(57.85–76.10)	(65.68–80.16)
HF norm (n.u.)	29.91	67.29	37.31	26.20
	(18.70–44.53)	(52.60–75.16)	(23.90–42.15)	(19.84–34.32)
LF/HF (n.u.)	2.34	0.49	1.68	2.82
	(1.25–4.35)	(0.33–0.90)	(1.37–3.18)	(1.91–4.04)
LF peak (Hz)	0.09	0.15	0.12	0.26
	(0.08–0.11)	(0.10–0.18)	(0.11–0.14)	(0.20–0.32)
HF peak (Hz)	0.30	0.36	0.67	1.77
	(0.26–0.35)	(0.30–0.42)	(0.51–0.77)	(1.35–2.51)
β (n.u.)	−0.82	−1.08	−0.68	−1.27
	(−1.28 to −0.36)	(−1.56 to −0.38)	(−0.90 to −0.32)	(−1.75 to −0.80)
SampEn (n.u.)	1.38	1.40	1.06	0.86
	(1.08–1.69)	(1.16–1.66)	(0.72–1.46)	(0.55–1.32)
SD1 (ms)	22.15	38.17	3.00	3.22
	(16.80–30.53)	(29.18–63.59)	(1.91–3.86)	(1.85–4.57)
SD2 (ms)	70.88	87.43	12.66	14.46
	(53.45–94.24)	(64.88–115.93)	(8.15–17.28)	(7.10–18.69)
α_1_ (n.u.)	1.20	0.86	1.21	1.17
	(1.03–1.33)	(0.71–0.95)	(1.11–1.34)	(1.06–1.25)
α_2_ (n.u.)	0.82	0.80	1.06	1.08
	(0.66–0.99)	(0.70–1.10)	(0.90–1.18)	(0.98–1.21)

*The human, rabbit, and mouse databases were preprocessed with moderate combined filtering (i.e., range plus moving average filter), and the dog database was preprocessed with the combined filtering with a moving average filter threshold at 40%. ^∗^For the human database we excluded files “16773,” “19093” and also parts of “19088,” “18177” because of inaccurate annotations in large parts of the recordings. Abbreviations: p, the number of 5-min segments for humans, dogs, and rabbits and 3-min segments for mice; n, the number of animals.*

Our third and main contribution is the creation of open source software (algorithms and user interface) for HRV analysis in different mammals. The software computes traditional HRV measures with parameters adapted to the mammal type. **Figure 3** provides an example of the PSD obtained from a 3 min mouse RR time series before and after atropine injection. It can be observed how the energy in the mouse HF band is eliminated by atropine and that this can be captured by the mammal-specific HF band defined in PhysioZoo. The software outputs standard HRV measures and standard visualization plots for analysis. Because the algorithms and user interface are both open source, it is easy to add additional HRV measures to the software or adapt it to any research-specific usage. In addition, the PhysioZoo software has a number of novel functionalities: it allows manual annotation of the signal quality and correction of misdetected R-peaks; it also allows the PhysioZoo interface HRV analysis to be performed on multiple segments (batch processing), thus making it possible to track changes in HRV measures over time for a given record.

Our fourth contribution is the discovery of a power law relationship between the mean SampEn and HR_m_ across mammals. Similarly, an allometric law with power ∼1/16 was found between the mean SampEn and BM_m_ of the different mammals (**Figure 6**). The results suggest that the complexity (in the sense expressed by sample entropy) of the heart rate increases with HR_m_ and decreases with BM_m_. This in turn suggests that the complexity of the heart rate decreases in smaller mammals. Beyond our analysis of SampEn, this finding opens an avenue for the study of HRV across species.

### Comparison With Existing Platforms

A number of commercial and open source HRV software products are available, the most popular ones being the PhysioNet HRV Toolkit (Mietus and Goldberger, 2017), Kubios (Niskanen et al., 2004; Tarvainen et al., 2014), and the R-HRV toolbox (Rodríguez-Liñares et al., 2008). In general, existing HRV software are (1) not tailored to the analysis of mammalian data other than that of humans; (2) do not allow manual annotation of the signal quality and correction of misdetected R-peaks; (3) do not provide a comprehensive open source code together with a functional user-friendly interface. The PhysioNet Toolkit is an open-source package, written in C, which includes standard HRV measures. Although this toolbox has the advantage of being compatible with all standard PhysioNet tools, it is not easy to install and use without programming skills. In addition, the source code does not include all standard HRV measures: some measures, such as the newly introduced fragmentation measures (Costa et al., 2017), are missing. The Kubios application includes all standard HRV measures and a user-friendly interface. It is likely the current most popular software for HRV analysis because the software is stable and does not require technical knowledge. However, the software is not open source, and thus its source code cannot be used for batch processing or any not readily available functionality. In addition, it does not allow tailored configuration files to be exported and reloaded for later analysis using the same user-specific parameters. R-HRV is a toolbox written in R. Like the PhysioNet HRV Toolkit, R-HRV presents a challenge for users without programming skills as it requires familiarity with the R environment. These limitations were addressed with the creation of the PhysioZoo platform.

PhysioZoo was implemented in MATLAB (The MathWorks, Inc., Natick, MA, United States). A standalone compiled version of the software is also available for users without a MATLAB license. Although Python has gained popularity in the past few years, particularly because it is open source and provides state-of-the-art open source libraries for machine learning, MATLAB still represents the reference platform for digital signal processing. Thus, we choose to implement PhysioZoo in MATLAB.

### Future Directions

The PhysioZoo platform was developed with a focus on ECG data analysis. However, the electrical potential that is measured on the body surface results from the integration of many biological system functions. Thus, the HR and its variability can be viewed as the result of the integration of individual cardiac pacemaker cell mechanisms (Yaniv et al., 2015a), the interactions among neighboring pacemakers in a cluster, and the influences of the external environment (e.g., neural influence) (Binah et al., 2013). As such, in order to better understand what mechanisms control the HRV, it is important to study the BRV at different levels of integration^4^: (1) the single pacemaker cell (BRV derived from the action potential firing rate); (2) sinoatrial node tissue (SAN), i.e., a cluster of interconnected pacemaker cells (BRV derived from the electrogram); (3) the isolated heart (BRV derived from the electrocardiogram, ECG). Recent studies have suggested that HRV is influenced by the two branches of the autonomic nervous system and by intrinsic properties of pacemaker cells (Yaniv et al., 2014, 2016). Although PhysioZoo can be used for BRV analysis at different levels, there are no existing recommendations on how to use the HRV measures for this type of data. We aim in the future to extend the capabilities of our software to enable working at different biological system levels; e.g., electrogram data from SAN tissues and action potential data measured using the patch clamp technique on sinoatrial node cells.

In addition, future development of PhysioZoo will include the incorporation of ECG morphological analysis tools (i.e., segmentation of the ECG cycles). Well known tools for Human ECG morphological analysis include the Glasgow program (Luo et al., 2004; MacFarlane et al., 2005) or the wavelet algorithm from Martínez et al. (2004).

### Limitations

Our databases and HRV measure parameters have been created/adapted for four of the main mammalian species used in cardiovascular research: humans, dogs, rabbits, and mice. However, contributions of electrophysiological data from other species such as pigs and sheep are welcome. The software configuration file can easily be updated to support the analysis of data from other mammals. It is also important to note that, contrary to the human ECG lead locations, the locations of the electrodes are not standardized for other mammals. Thus, the morphology of the mammalian ECG contained in the PhysioZoo database may vary depending on the contributor of the data.

From our experience with the PhysioZoo databases, we recommend that the sampling frequency of the ECG in mice be at least 1 kHz. Indeed, because of the high HR of mice, the R-peaks are usually represented using only a few samples, thus rendering the localization of the R-peaks challenging. In addition, despite using the maximal precision available when exporting the rabbit data, we noted that the quantization of the original files was not adequate. Although we could accurately detect the R-peak for HRV analysis, we do not recommend using the PhysioZoo rabbit ECG data for morphological analysis.

## Conclusion

The PhysioZoo initiative enables the standardization and reproducibility of HRV analysis in mammalian models through its open source code, freely available software, and open access databases. It is intended to stimulate current research and new investigations in mammalian HRV analysis. We made available the source code and software on physiozoo.com and the databases on physionet.org.

## Data Availability

The datasets for this study can be found on PhysioNet (https://www.physionet.org/). The source code for the PhysioZoo software can be found on the PhysioZoo website https://physiozoo.com/ and accessible on GitHub at https://github.com/physiozoo/physiozoo.

## Author Contributions

JB and YY conceived and designed the research. AR, AA, JB, EK, IW-B, and OS implemented the source code, interfaced and formatted the databases. JB drafted the manuscript. AR, JB, and YY wrote the supplement. JB and YY edited and revised the manuscript. JB, YY, AR, OS, IW-B, AA, and EK approved the final version.

## Conflict of Interest Statement

The authors declare that the research was conducted in the absence of any commercial or financial relationships that could be construed as a potential conflict of interest.

## Footnotes

1 https://www.physionet.org/tutorials/hrv-toolkit/

2 https://www.physionet.org/physiotools/dfa/

3 https://physionet.org/physiotools/mse/

4 In the context of this paper HRV refers to the analysis of the beat-to-beat interval variations from *in vivo* ECG data and BRV to a more generic term used to denote the study of the beat-to-beat interval variations from any electrophysiological signal (e.g., sinoatrial note cell, tissue and whole vivo-heart).

## Abbreviations

BRV: beating rate variability
HRV: heart rate variability
NN: filtered RR interval
NSR: normal sinus rhythm
PSD: power spectral density
RR: beat-to-beat interval derived from the ECG signal.
