# Supplementary material for: PhysioZoo: A Novel Open Access Platform for Heart Rate Variability Analysis of Mammalian Electrocardiographic Data
Source: Front Physiol. 2018 Oct 4;9:1390. doi: 10.3389/fphys.2018.01390 (PMC6180147; doi:10.3389/fphys.2018.01390)
Supplement: Supplementary file 1 [file Data_Sheet_1.pdf]

PhysioZoo: a novel open access platform for heart rate variability analysis of mammalian electrocardiographic data

Running title: PhysioZoo for mammalian HRV analysis

Joachim A. Behar[#,*,1], Aviv A. Rosenberg[#,1,2], Ido Weiser-Bitoun[1], Ori Shemla[1], Alexandra Alexandrovich[1], Eugene Konyukhov[1], and Yael Yaniv[*,1]

[1] Biomedical Engineering Faculty, Technion-IIT, Haifa, Israel

[2] Computer Science Department, Technion-IIT, Haifa, Israel

# Co-first authors

* Corresponding authors

Supplements

Supplement 1: This supplement is divided into three parts: (1) additional information related to the R-Peak detector configuration in PhysioZoo, RR interval preprocessing, and parameters of the HRV measures used in the core publication (Content items 1-3); (2) background theory for the HRV measures implemented (Content item 4); (3) some additional tables providing more details about the databases used in this paper (Content item 5).

Supplement 2: This supplement provides more details about the procedure for comparing the HVR measures from PhysioZoo and the HRV algorithms available on PhysioNet for the human ECG data.

1. R-Peak detection

1.1 Evaluation on standard human databases

To quantify the accuracy of a peak detector, the detected peaks must be compared to manually corrected reference peak annotations. The tolerance window (denoted w) is defined as the time interval between a reference annotation and a detected peak during which they are still considered to be a match. If the detected peak and the annotated reference peak are in a range of $\pm w$ of each other, then the detected peak is considered to be a true positive (TP). If there is no detected peak within $\pm w$ of a reference peak, then this is considered a false negative (FN). Finally, if a detected peak does not fall within $\pm w$ of a reference peak, then it is considered to be a false positive (FP). This is illustrated in Figure S1.

To make sure *rqrs* performs robust R-peak detections (at least as accurate as the Physionet *gqrs* algorithm), the algorithm was tested on the updated 2014 PhysioNet Challenge training set [1]. This set contains 200 ECG recordings, some of which are very challenging for R-peak detection algorithms due to noise and artifacts [1]. It was previously used to assess various detector algorithms, including *gqrs* [2], so its performance is known on this set. All ECG recordings in the set have reference, manually corrected, R-peak annotations.

A comparison between the results of *gqrs* and *rqrs* on the PhysioNet 2014 Challenge database can be seen in Table S1. For *gqrs* the expected results are nearly identical to the results reported in Johnson et al. [3]. The *rqrs* algorithm performs similarly to *gqrs*, with a slightly better overall score, arguably due to the fact that it finds the R-peaks themselves, increasing the chance that a reference annotation will be within the comparison window around the detection.

	Mean		Gross		
	Se	*PPV*	*Se*	*PPV*	*F1*
gqrs	93.73	92.57	94.23	90.93	92.55
rqrs	92.85	93.68	93.15	93.08	93.12

Table S1: Performance comparison of *gqrs* and *rqrs* on the PhysioNet 2014 Challenge database. The gross values were calculated over all detections and annotations in the set, without averaging each record individually.

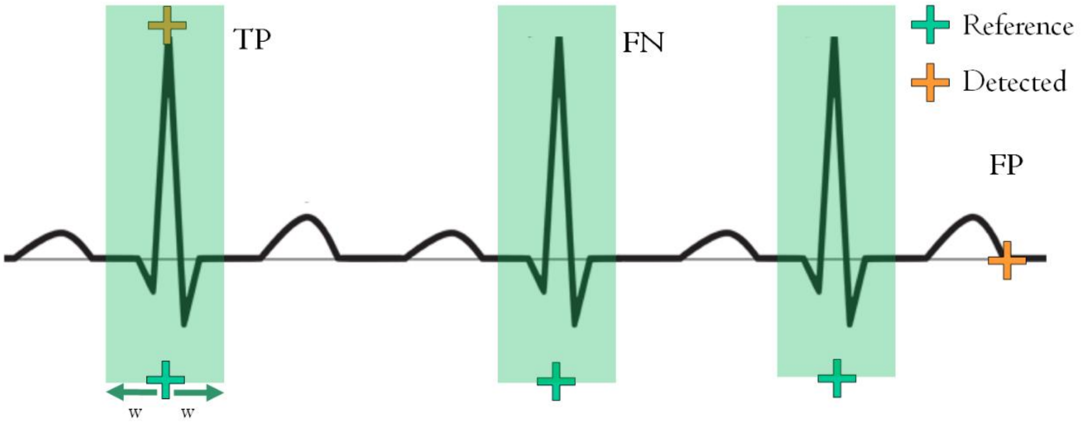

Figure S1: Performance evaluation of a peak detector. w: tolerance window, TP: true positive, FN: false negative and FP: false positive.

1.2 Adaptation of the parameters for other mammals

In order to adapt the R-peak detection algorithm to ECG data from each mammal, several parameter modifications were required; HR_m, the typical heart rate of the mammal, taken as being the mean HR; QS_m, the typical QRS duration, taken as being the mean QRS duration; QT_m, the typical QT interval duration, taken as being the mean QT duration; RR_{min}, the minimum RR interval; RR_{max}, the maximum RR interval; QRS_a the typical QRS peak-to-peak amplitude; and QRS_{amin}, the minimum QRS peak-to-peak amplitude.

For the dog parameters, we used the data reported by Hanton and Rabemampianina [4], who measured ECG parameters of beagles. We used the parameters obtained by measurements from 658 healthy male Marshall dogs placed in standing position and gently restrained. Needle electrodes were inserted subcutaneously. According to their measurements and calculations, the HR_m was 109.5 bpm, the QS_m duration was 42 msec, and the QT_m was 199 msec. QRS_a and QRS_{amin} were obtained from Cintra et al. [5], who studied the influence of opioid administration on sixteen healthy adult female mongrel dogs divided into two equal groups. Electrodes were placed on the limbs of the dogs. We used the mean and min 'spread R wave' amplitudes of one of the groups (8 dogs) before the administration of any drug as an approximation for QRS_a and QRS_{amin}. The 'spread R wave' was 1.12±0.34 mV. Thus, we set QRS_a to be: 1120 μV and QRS_{amin} to be 100 μV (taken as being three standard deviations from the mean value).

For the rabbit parameters, we used the measurements reported by Lord et al. [6], who measured the electrocardiograms of 46 healthy domestic pet rabbits with normal sinus rhythm. Standard crocodile clip electrodes were used (with their teeth bent outwards) and applied to a skin fold. According to their measurements, the HR_m was found to be 264 bpm, and the QT_m was 0.12 sec. The QS_m duration was 0.04 sec, and for conservative reasons we used their lower range, 0.02 sec. For the R-wave amplitude we used the measurements reported by Chapel et al. [7], who measured the electrocardiogram of 44 Netherland dwarf rabbits (28 intact females, 10 intact males and 6 neutered males) without respiratory or cardiac diseases. Toothless alligator clip electrodes were used for the measurement. They found that the mean R-wave amplitude measured in the sternal recumbence position was 0.294±0.06 mV. We took this R-wave amplitude as an approximation for the QRS_a, and we calculated the QRS_{amin} as being three standard deviations from the mean value, or 0.114 mV.

For the mouse parameters, we used the measurements reported by Sysa-Shah et al. [8], who measured the electrocardiograms of 695 wild type B6SJLF1/J mice by subcutaneous electrodes. According to their measurements and calculations, the HR_m was found to be 608 beats per minute and the QS_m was 7.18 msec. R-wave amplitude was 1.09 mV. We took this R-wave amplitude as an approximation for the QRS_a amplitude. The QT_m interval was calculated from the QT_c using the formula reported in Mitchel et al. [9], and it was found to be 0.03 sec. QRS_{amin} was estimated as 370 μV by subtraction of two standard deviations of the R-wave amplitude from the mean R amplitude (which represent 95.45% of the values, assuming a normal distribution of the R-wave amplitude).

For dogs, rabbits and mice, the RR_{min} and RR_{max} were calculated as being 75% and 125% of the typical RR range reported by the Research Animal Resource Center, University of Wisconsin—Madison [10]. Indeed, RR_{min} and RR_{max} correspond to the extreme values that are tolerable for a given mammal and thus more flexible than the typical range. For the dogs, we set RR_{max} to be 140% of the RR range upper bound reported by the Research Animal Resource Center, University of Wisconsin—Madison [10]. This is because the reported range is provided for beagles, and thus we took a larger RR_{max} to account for bigger dog species (while noting that, ideally, the R-peak detector should be fine-tuned to fit the specific breed).

2. RR interval preprocessing

Numerous methods exist to quantify HRV by looking at the beat to beat interval length variations (refer to Malik et al. [11] for an overview). The basic interval lengths can be obtained by measuring the time differences between consecutive R-peaks (RR intervals). However, by definition, only beats resulting from normal sinus node depolarizations (i.e., not arrhythmic, paced, ventricular, etc.) should be used for HRV metric calculations. The intervals between such normal beats are referred to as NN intervals.

We would like to reject so-called ectopic beats, a type of premature beat that is quite common in ECG signals and often originates from the atrioventricular node or pacemakers in the ventricles due to a blockage of the depolarization signal from the sinoatrial node (SAN) [12]. Rejecting ectopic beats will allow us to isolate the contribution of the SAN to the HRV. In addition to ectopic beats, ECG signals generally contain various artifacts caused, e.g., by movement of the subject or by the recording equipment. Such artifacts should ideally be removed prior to any attempt at HRV analysis.

In order to obtain the NN intervals, RR intervals are found using an ECG R-peak detection algorithm, and then a preprocessing step (either manual or automatic) is performed to filter out suspected ectopic beats and artifacts.

It should be noted that ECG and HRV analysis methods, as described in the literature, are usually designed for use with human ECG data and thus might need to be adapted when considering data from other mammals.

2.1 Methods for RR interval preprocessing

An RR interval is the time difference between two adjacent R-peaks in an ECG signal. More specifically, given a series of N R-peaks timestamp $t_0, \dots, t_{N-1}$, the corresponding RR interval time series is defined as

(1) $$RR(t_i) = t_{i+1} - t_i \,, \forall\, i \;\in 0,1, \dots, N-1.$$

Note that the RR interval time series is defined at the R-peak detection times only, and each interval at a specific peak time is the time from that peak to the next [11].

We would like to find the subset of these intervals whose corresponding beats are normal sinus beats. Because there is no way to know with absolute certainty whether an ECG peak originates from the SAN, it will be enough to greatly reduce the likelihood of having non-NN intervals in the preprocessed data. One way to do this is to improve the ECG R-peak detector so that it detects fewer non-SA peaks in the first place. Another approach is to analyze the RR interval time series and discard suspect intervals based on some criterion, typically an instantaneous increase by more than 20% in the RR interval with respect to surounding RR intervals [12]. We focused on the second approach, aiming to analyze the RR intervals to remove those suspected of not being NN. Multiple preprocessing steps were implemented to remove suspect intervals. The preprocessing steps used here are based on works by Brennan et al., Clifford, Moody, and Piskorski and Guzik [12]–[15], and also on the preprocessing performed by the PhysioNet s

a. Range-based filtering (RBF). RR interval duration inversely relates to the instantaneous heart rate:

(2) $$IHR(t) = \frac{60}{RR(t)}$$

where $RR(t)$ is an RR interval, in seconds, measured at time t, and $IHR(t)$ is the instantaneous heart rate in bpm at time t. This means that there are upper and lower bounds on the RR intervals that can be

obtained from the physiological limits of the heart rate. For example, if we assume the instantaneous heart rate will be in the range of 40–187.5 bpm, then the range of RR interval values must be 0.32–1.5 seconds; intervals not within this range can thus be discarded as non-NN.

b. Moving average filter (MAF). In some cases the RR interval time series can contain intervals that are very short or very long when compared to their neighboring intervals and can appear as pronounced “spikes” when the RR intervals are plotted. While beat-to-beat variability is what we are looking for, these spikes indicate a significant change in RR interval duration between adjacent intervals or relative to neighboring interval values. This could, in turn, indicate that at least one of the beats in the interval was ectopic or perhaps even a false detection. To remove such intervals, a moving average filter filter is passed over the RR interval time series. The average in the window is calculated without the sample in the center of the window. If the central sample’s value exceeds (in absolute terms) the window average by some percentage, that sample is removed. We decided to use a window size of 21 samples (10 samples on each side of the central sample) and filtered out the sample if its value exceeded 20% of the window’s average (default option corresponding to the ‘medium’ prefiltering level). The threshold and window size were chosen to be identical to the values used by the PhysioNet HRV toolkit [17].

c. Quotient filter (QF). This filter removes intervals that vary by more than some percentage q from either the next or the previous interval [15]. It is conceptually similar to the sliding window average filter but much more local. For each interval, the filter examines the ratio between it and the previous and next intervals. If this ratio exceeds a q percent change, the interval is discarded. More specifically, we define $r = \frac{q}{100}$ (so $0 < r < 1$). The filter rejects the interval at time t_i, $RR(t_i)$, if at least one of the following conditions is true:

(3) $$\frac{RR(t_i)}{RR(t_{i+1})} < 1\text{-r, or } \frac{RR(t_i)}{RR(t_{i+1})} > 1\text{+r}$$

(4) $$\frac{RR(t_{i+1})}{RR(t_i)} < 1\text{-r, or } \frac{RR(t_{i+1})}{RR(t_i)} > 1\text{+r}$$

For humans, a value of $q = 20\%$ was shown to work well for removing non-physiological intervals in data from heart-failure patients [15]. The same value ($q = 20\%$) was used as a default option corresponding to the ‘medium’ prefiltering level. This filter is useful for removing strong local noise (very short or long intervals) due to its aggressiveness.

Figure S2 and Figure S3 show a short segment of ECG that contains a noisy region, the corresponding RR interval time series in that segment with suspected non-NN intervals, and the resulting filtered intervals. The ECG signal used was fantasia/f1y06 from the PhysioNet Fantasia database [16], [18].

Prefiltering with the PhysioNet HRV Toolkit vs. PhysioZoo

The PhysioNet HRV Toolkit [17] implements a prefiltering step that is equivalent to first applying the RBF and then the MAF. In our implementation of the MAF, we used a zero phase filter by processing the input data in both the forward and reverse directions (MATLAB *filtfilt.m* function). This filter minimizes start-up and ending transients by matching initial conditions. It also allows us to keep the length of the original input data. This is different from the PhysioNet HRV toolkit [17], which removes a number of NN data samples equal to the filter window length (i.e., 41 datapoints if we use the traditional window length). which might be limiting when studying 5 min windows. As an example, for a human RR time series with a HR at 60 bpm, removing 41 datapoints will correspond to removing 41 sec of the original window length to account for the initialization of the HRV Toolkit filter, thus making the effective window 4 min and 19 sec long (and thus closer to 4 min than to 5). This is illustrated in Figure S4.

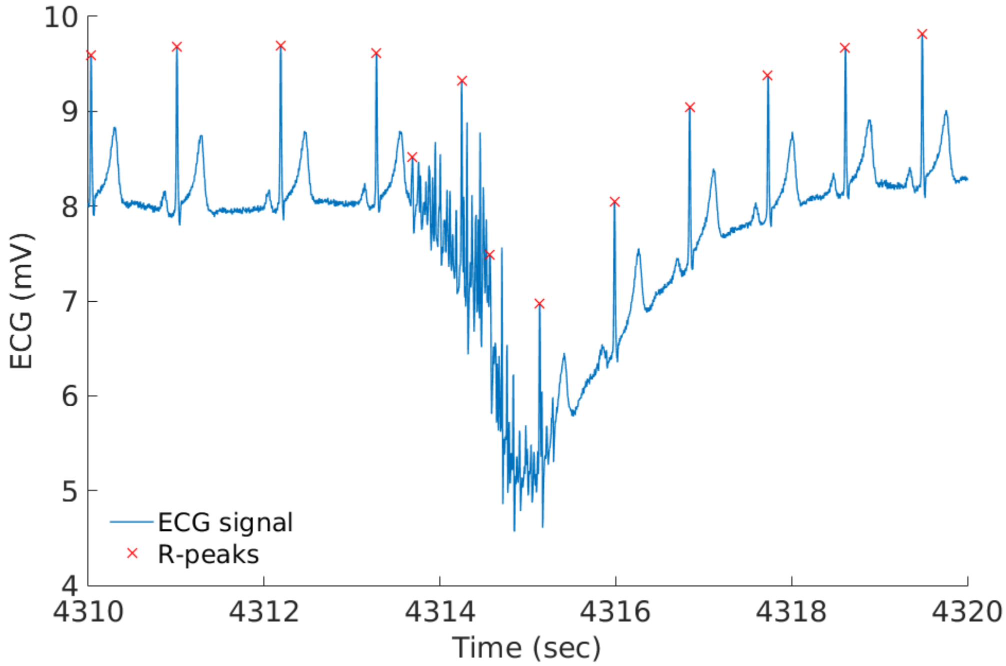

Figure S2: short ECG segment showing R-peaks. Visual inspection shows a relatively long interval starting at 4311 sec, and a noisy region at 4313–4316 sec. RR interval filtering of fantasia/f1y06 [16].

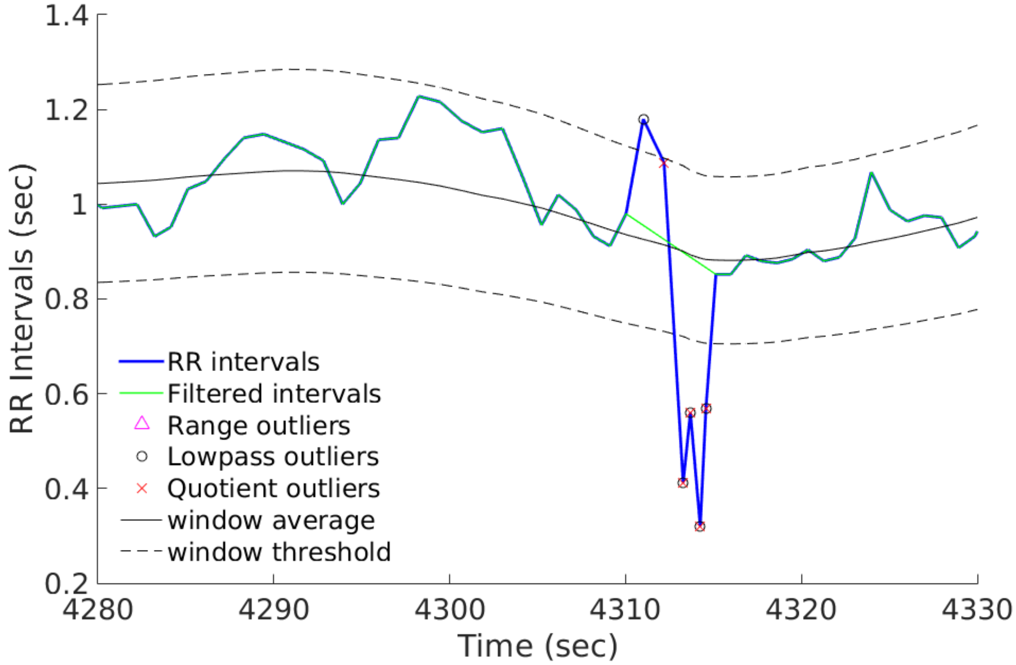

Figure S3: RR interval time series before and after filtering, showing the intervals that were flagged for removal relative to the filter used. We can see a series of four very short intervals starting at 4313 sec, which correspond to a noisy region in the corresponding ECG (see Table **S2**). In addition, we can see the quotient filter rejection of the interval at 4312, due to the large change relative to the next interval. We can also see the moving average filter rejection of the interval at 4311 due to it being more than 20% longer than the average in its neighborhood. RR interval filtering of fantasia/f1y06 [16].

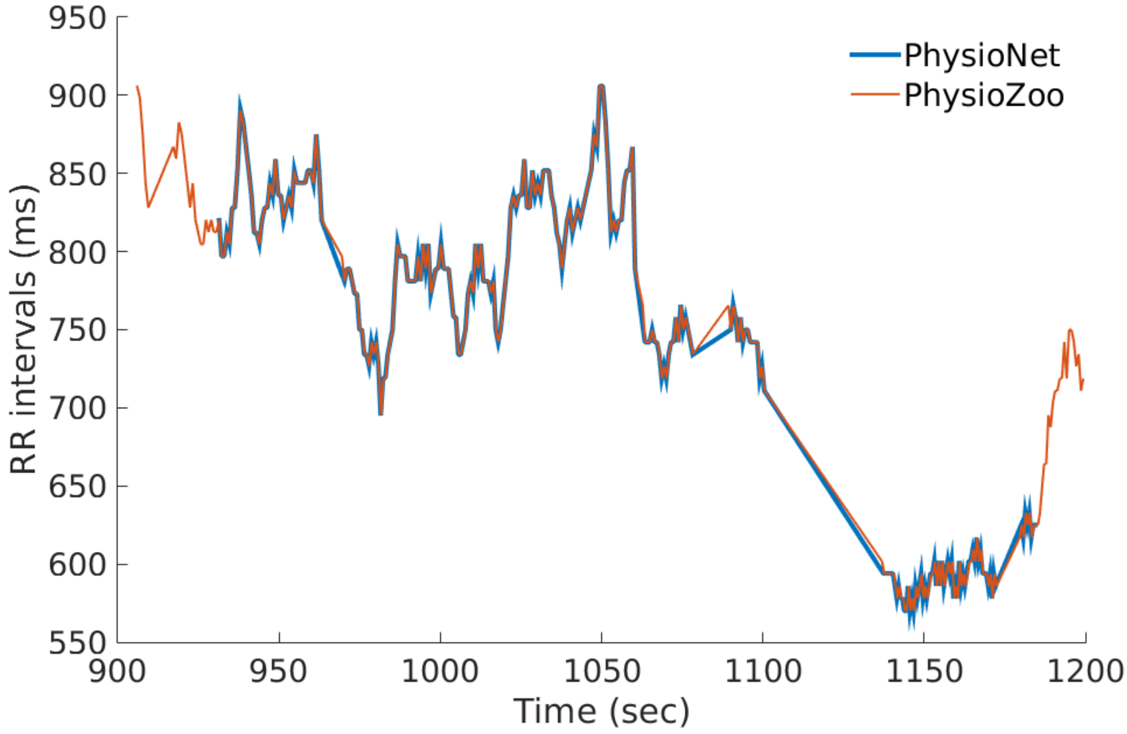

Figure S4: Example of the prefiltering step using the PhysioNet HRV toolkit and the PhysioZoo combined filter (range plus moving average filter) on a five minute (i.e., 300 second) window. As can be seen, the PhysioNet HRV toolkit filter removes ~40 seconds of the data due to the border effect, thus making the effective window closer to 4 min than to rather than 5 min, which will in particular affect the minimal resolvable frequency.

3. Heart rate variability measures

HRV measures are traditionally broken down into three categories: time, frequency, and nonlinear measures.

3.1 Power spectral estimation

In healthy humans, the frequency of the power spectral density performed on a 5-minute long RR time series is traditionally divided into three main bands [11]: the very low frequency (VLF) band, the low frequency (LF) band and the high frequency (HF) band.

Three different power spectral estimation techniques are traditionally used for HRV analysis: Welch's method, the auto-regressive model, and the Lomb method. The Lomb method has the advantage of not requiring resampling of the RR time series. However, it is prone to aliasing; Figure S5 shows an example of a power spectrum from a mouse NN time series. The aliasing that occurs with the Lomb estimation method is clearly visible starting at around 3 Hz (circled in red). This is due to the limited upper bound that can be resolved by the Lomb PSD estimation method while still satisfying the Nyquist criterion. In addition, the upper bound the Lomb method can resolve will depend on the average NN interval in the recording (and is thus variable). Because of these limitations, we decided not to implement the Lomb PSD estimation method in PhysioZoo.

For the AR model, we set the AR order manually for each species based on whether we could visualize the VLF, LF and HF modes (i.e. 3 modes) with the AR spectrum.

Important considerations with the Welch PSD estimation

- **Frequency resolution:** this is defined by $\Delta f = \frac{1}{T}$, where T is the length of the selected window. The longer the window, the thinner the resolution and the lower the minimal resolution frequency.
- **Resampling:** necessary because the RR time series is unevenly sampled. The choice of the resampling rate is important because it will define the maximal resolvable frequency. Thus, the resampling rate must be at least twice the maximal frequency we want to resolve to satisfy the Nyquist-Shannon sampling theorem.

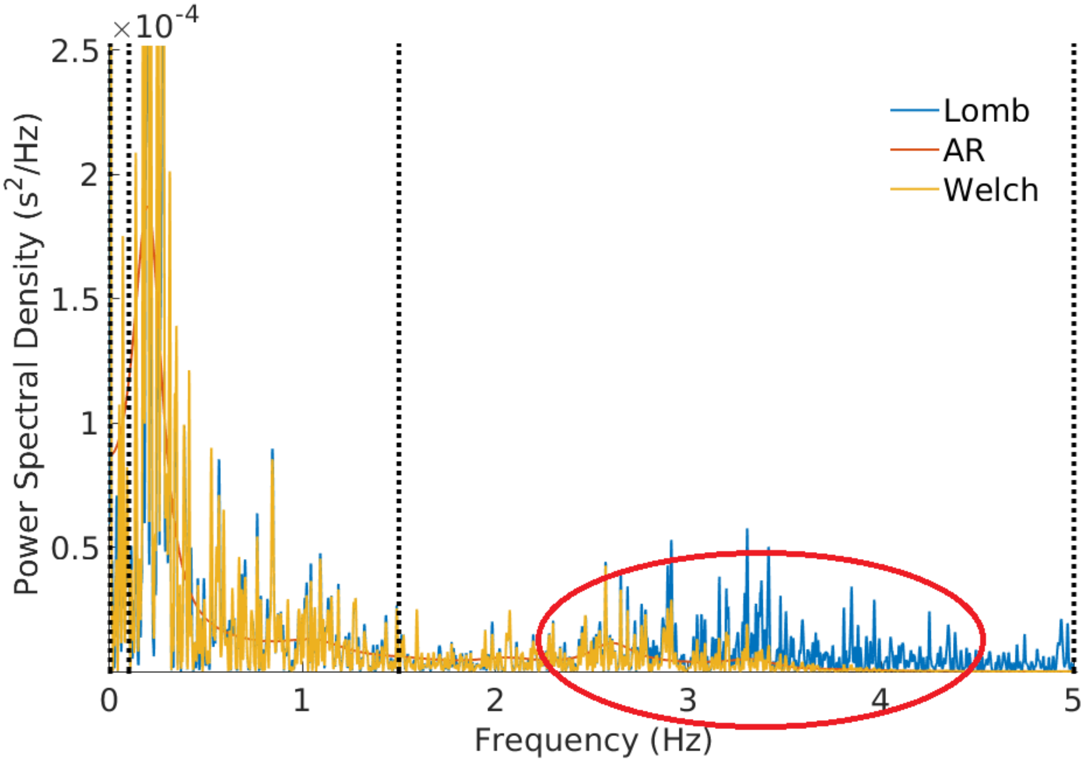

Figure S5: Example of power spectrum from a mouse NN time series. The aliasing that occurs with the Lomb estimation method is clearly visible, starting around 3 Hz (circled in red). This is due to the limited upper bound that can be resolved by the Lomb PSD estimation method while satisfying the Nyquist criterion.

3.2 Detrended Fluctuation Analysis

Detrended Fluctuation Analysis (DFA) [19] can be used in the context of HRV to study the fractal like behavior of the time series [20]. In DFA analysis of the NN time series, the fractal behavior is divided into two parts: the low-scale slope and the high scale slope. The "crossover" point [20] is typically n ~ 16 beats and is believed to be related to respiratory sinus arrythmia. This "crossover" could technically change across species. We looked for a relationship between the typical heart rate (HR_m) of different mammals and their typical breathing rate (BR_m) by using normative data provided by the University of Wisconsin–Madison [10]. We found a linear relationship: $BR_m = 0.26 * HR_m$ with $R^2 = 0.89$ (Figure S6). This is in accordance with two published allometric laws [21], [22]: HR_m and the body mass (BM) have a power law relationship, with power ~ -1/4, and $BR_m$and BM have a power law relationship, with power ~ -1/4. This linear relationship also implies that the number of beats in one breathing cycle is the same for all species. Thus, we do not expect the DFA "crossover" point to change across mammals. Exploring this through our databases revealed that the "crossover" point n = 16 is suitable for all studied species.

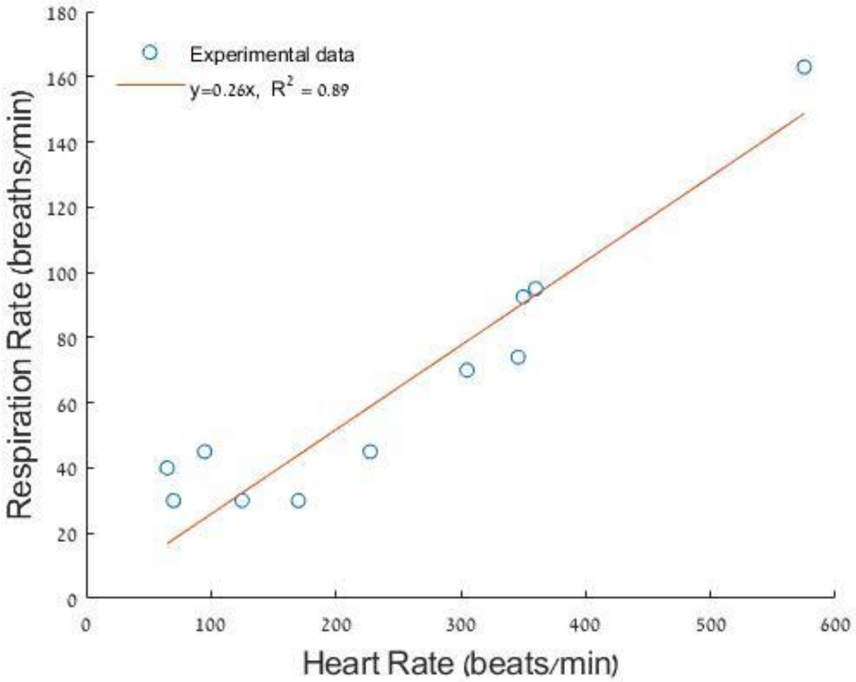

Figure S6: Typical breathing rate as function of heart rate for 11 types of mammals (laboratory mouse, gerbil, laboratory rat, Syrian hamster, guinea pig, laboratory rabbit, cat, dog, swine, cow, sheep). The typical BR and HR of these animals were scattered and the trend line (forced to pass through the origin) was calculated (SE=0.01) and plotted. The linear relationship between BR and HR suggests that the definition of α_1 and α_2 in DFA can be preserved for different mammals.

3.3 Benchmarking HRV time measures

The MIT NSR databases (NSRDB and NSR2DB, Table **S2**) were used as benchmarks to compare the HRV measures from PhysioZoo and the HRV algorithms available on PhysioNet (HRV Toolkit[1], MSE[2] and DFA[3]). All records were included in the comparison and each record was split into 1-hour long windows. We report the mean (μ) and standard deviation (σ) of the HRV measures over the NSR databases using the two toolboxes (PhysioZoo/PhysioNet). We also report the root mean square error (RMSE) and the normalized root mean square error (NRMSE) of the comparison between the two toolboxes. We defined the NRMSE as being the RMSE normalized by the mean of the HRV measures computed using the PhysioNet source code (used as the reference). We compared the Lomb function available from the PhysioNet HRV Toolkit which uses a rectangular window against the PhysioZoo Lomb implementation while using a rectangular window in order to perform an objective comparison. However, we note that our final implementation choice in PhysioZoo was to choose the default spectral estimation methods to be the Welch/AR methods (because of some Aliasing we noted with the Lomb's method, Figure S5) and using a Hamming window in order to limit spectral leakage. More details on the procedure for benchmarking between the toolboxes are provided in the supplementary document: `Procedure for HVR benchmark'. Table S3 demonstrates the very low NSMSE obtained for all the measures. The residual differences may be due to errors resulting from the difference in rounding between code in MATLAB (PhysioZoo) and C (PhysioNet). However, the errors are minor and will not affect HRV analysis.

Database	Type	Duration	Age Range	Records
NSRDB	NSR	24h	20–50	18
NSR2DB	NSR	24h	28–76	54

Table S2: Databases used for HRV benchmark.

	**PhysioNet**		**PhysioZoo**			
	**μ**	**σ**	**μ**	**σ**	**RMSE**	**NRMSE**
NN_RR	0.99976	4.44522E-05	0.98917	0.02506	0.02718	0.02718
AVNN	826.565	144.092	826.567	144.093	0.02486	3.008E-05
SDNN	74.2688	28.1917	74.2752	28.1900	0.09370	0.00126
RMSSD	30.6062	18.0546	30.6096	18.0540	0.10435	0.00341
pNN50	9.79078	12.5054	9.79128	12.5062	0.00749	0.00077
Total power	6775.94	5817.75	6610.87	5617.40	384.084	0.05668
VLF	1940.55	1715.51	1938.23	1714.97	98.6525	0.05084
LF	794.073	725.906	789.574	716.259	20.8454	0.02625
HF	390.314	717.814	391.553	709.615	30.5741	0.07833
LF/HF	3.78348	2.70846	3.69184	2.57410	0.22186	0.05864
MSE	1.13773	0.31667	1.13773	0.31668	0.00030	0.00026
DFA	-1.19930	0.42082	-1.19671	0.42563	0.01391	0.01160

Table S3: Validation of some standard HRV measures included in PhysioZoo against their implementation in the PhysioNet HRV toolkit. μ: mean; σ: standard deviation; RMSE: root mean square error; RMSE: normalized root mean square error. For MSE and DFA, the values estimated at each scale (1-20 for MSE and 4-64 for DFA) were used for comparison.

1 https://www.physionet.org/tutorials/hrv-toolkit/
2 https://www.physionet.org/physiotools/mse/
3 https://www.physionet.org/physiotools/dfa/

4. Background theory for HRV measures

4.1 Time domain measures

Common measures

The most straightforward HRV analysis methods rely on linear time-domain statistics. For $i = 0, \ldots, N-1$, we will denote t_i as the beat timestamp and and $NN(t_i)$ as the NN interval beginning at time t_i. The following well-known measures were implemented. SDNN: The standard deviation of all NN intervals

(5) $$SDNN = \sqrt{\frac{1}{N-1}\sum_{i=0}^{N-1}(NN(t_i) - \overline{NN})^2}$$

where $\overline{NN}$ is the mean NN interval time.

RMSSD: Root mean square of successive differences (SD) of NN intervals

(6) $$RMSSD = \sqrt{\frac{1}{N-1}\sum_{i=0}^{N-1}(NN(t_i) - NN(t_{i-1}))^2}.$$

pNN50: The percentage of NN intervals which differ by at least 50 ms from their preceding interval

(7) $$pNN50 = \frac{1}{N-1}\sum_{i=1}^{N-1} I_i^{50}$$

where I_i^{50} is the indicator

$$I_i^{50} = \begin{cases} 1, & |NN(t_i) - NN(t_{i-1})| > 50[ms] \\ 0, & else \end{cases}.$$

In particular, the $pNN50$ measure was derived from the original study of Ewing et al. [23]. The authors introduced the $NN50$ measure, defined as the mean number of times per hour in which the change in the NN intervals exceeded 50 ms. The authors suggested this measure to assess parasympathetic (vagal) activity from 24-hour ECG recordings. Later, Bigger et al. [24] introduced the $pNN50$ measure. The $pNN50$ is a member of the broader class of statistics, $pNNxx$. A study on optimizing the xx value was conducted by Mietus et al. [25].

Fragmentation measures

In their recent work, Costa et al. [26] introduced four new HRV measures designed to quantify the degree of sinus rhythm fragmentation when considering short-term HRV. The four measures were defined as:

- PIP: the percentage of inflection points in the NN interval time series, defined as the percentage of zero-crossing points in the increment time series. A point, t_{N_i} is defined as an inflection point if:
$$\Delta NN_i * \Delta NN_{i+1} \leq 0.$$
- IALS: the inverse of the average length of the acceleration/deceleration segments. An acceleration/ deceleration segment is a sequence of NN intervals between consecutive inflection points for which the difference between two NN intervals is < 0 and > 0, respectively.
- PSS: the percentage of short segments. This quantity is defined as the complement of the percentage of NN intervals in acceleration and deceleration segments with three or more NN intervals.
- PAS: percentage of NN intervals in alteration segments. An alternation segment is a sequence of at least four NN intervals, for which heart rate acceleration changes sign every beat. Such sequences follow an “ABAB” pattern, where “A” and “B” represent increments of the opposite sign.

4.2 Frequency domain measures

Another HRV analysis approach is to perform power spectral analysis of the NN time series to estimate the frequency content of the NN time series fluctuation, i.e., the contribution of each frequency component to the signal's power. To do this, the power spectral density of the NN interval time series needs to be estimated:

(8) $$\{t_i, NN(t_i)\}_{i=0}^{N-1},$$

which is essentially a finite realization of a random process. A number of methods exist for spectral estimation of random processes. They can be broadly categorized as parametric and nonparametric [27] Parametric methods rely on a predetermined model of the process producing the samples, usually an autoregressive moving average model. The model parameters are estimated from the sampled data, and then the spectrum can be calculated with arbitrary precision based on the model [28]. Nonparametric methods compute the spectrum directly, usually using Fourier-based analysis of the data itself and are more severely limited by the quantity and quality of the data.

Nonparametric spectral analysis

A major nonparametric spectral analysis technique is to use a periodogram to estimate the spectrum of a random process. The periodogram $\hat{S}_X[k]$ of a time-series $x[n]$ of length *N* sampled at f_s Hz is defined asthe squared magnitude of the Discrete Fourier Transform (DFT) of the signal:

(9) $$\hat{S}_X[k] = \frac{1}{N}|X[k]|^2 = \frac{1}{N}\left|\sum_{n=0}^{N-1} x[n]e^{-\frac{j2\pi kn}{N}}\right|^2,$$

where k is a discrete frequency index such that the corresponding physical frequency in Hz is:

(10) $$f(k) = k \cdot \frac{f_s}{N}, k = 0,1,\ldots,\frac{N}{2} - 1.$$

We only consider $\frac{N}{2}$ values of k because we are dealing with real-valued input signals. The periodogram, while simple, is not a consistent estimator for the PSD—it does not converge to the actual PSD as the number of samples increases. It moreover suffers from spectral leakage due to rectangular windowing in the time domain, which is equivalent to a frequency domain convolution with a $sinc$ function [28]. A more robust version of the periodogram, which suffers less from these drawbacks, is the Welch periodogram [29], defined as follows by Porat and Boaz [27]:

(11) $$\hat{S}_X[k] = \frac{1}{L}\sum_{l=0}^{L-1}\{\frac{1}{N}\left|\sum_{n=0}^{N-1} w[n] \cdot x[n + \frac{0.5}{N}]e^{-\frac{j2\pi kn}{N}}\right|^2\}.$$

To calculate Welch's periodogram, the input signal is split into L overlapping segments, each of length *N*. Each segment is multiplied by a window function $w[n]$ and its DFT is calculated. The DFT of all segments is then averaged. Note that the $\frac{0.5}{N}$ term creates a 50% overlap between segments. Welch's method sacrifices spectral resolution for smoothness obtained by averaging multiple shorter DFTs and reduces spectral leakage by using a non-rectangular window function. An example of the Welch periodogram performed on a 5 minutes Human RR time series is shown in Figure S7.

Parametric spectral analysis

Parametric spectral estimates are based on the modeling of random processes. The idea is to assume the signal is the output of a causal and stable linear digital filter *B(z)* driven by a white noise input process *v[n]* with variance σ_v^2. The random process can then be represented by the filter parameters and the variance of

the white noise [28]. The most commonly used parametric model is the autoregressive (AR) model because it allows for relatively simple calculation of model parameters based directly on the sample autocorrelations of the data sequence itself (Yule-Walker method [30], [31]). AR models have been commonly employed for spectral estimation of RR interval time series [11], [13]. An AR model assumes the signal is a linear combination of p of its previous values, i.e., it is of the form

(12) $$x[n] = v[n] - a_1 x[n-1] - a_2 x[n-2] - \cdots - a_p x[n-p],$$

which is the difference equation corresponding to an all-pole digital filter of order p with a transfer function:

(13) $$B(z) = \frac{1}{1 + a_1 z^{-1} + a_1 z^{-2} + \cdots + a_p z^{-p}}.$$

For such a filter, the PSD can be computed directly from the transfer function [28]:

(14) $$S_X(w) = \sigma_v^2 |B(z = e^{jw})|^2 .$$

This allows analytical calculation of the PSD at any precision.

Many cardiologists prefer to use the AR model [32], because it is easier to visually identify the characteristic LF and HF peaks with this representation versus the non-parametric methods. The AR approximation gets closer to the Fourier spectrum as the model order increases [32]. An example of the AR model for PSD estimation is shown for a 5 minutes Human RR time on Figure S7.

Spectral analysis assumptions

An important assumption for spectral estimation techniques of random processes is the stationarity of the process. This means that the statistical properties of the signal (mean, variance and higher-order moments) should be time-invariant. In practice it is satisfactory to consider wide-sense stationary signals where only the mean and variance are constant with time. This follows from the Wiener-Kinchine theorem, which states that wide-sense stationarity is a sufficient requirement for computing the power-spectral density $S_X(f)$ of a stochastic process *x(t)* from the Fourier transform of its autocorrelation $R_X(\tau)$ [33]:

(15) $$S_X(f) = \int_{-\infty}^{\infty} R_X(\tau) e^{-j2\pi f t} d\tau.$$

The use of 5-minute data segments recorded at rest has therefore become the standard for HRV analysis in the frequency domain [11]. It is assumed that the statistical properties of the interval time series are sufficiently stationary in these short segments.

In addition to the stationarity assumption, most spectral estimation techniques assume a uniformly sampled time series input. In our case, the NN interval time series is by definition not sampled uniformly. To obtain a nonparametric spectrum estimation of the NN interval time series without the need for resampling, the Lomb-Scargle periodogram [34], [35] has been previously used. This method is well known, and is derived by solving a least-squares optimization problem of fitting a series of sinusoids to the given data. The Lomb-Scargle periodogram was shown to provide favorable results on NN interval data [13]. For other spectral estimation techniques, resampling to a constant sampling frequency is required. Cubic spline interpolation was previously shown to work well for resampling NN interval data [14].

Spectral bands

Spectral analysis of the NN intervals reveals that periodic components are present in the interval time series at frequencies below the HR [36]. In healthy humans, the frequency content of the interval lengths in a 5-minute recording is typically analyzed in three main frequency bands (ranges) [11]:

- The high frequency (HF) band, from 0.15 to 0.4 Hz. Characteristically, a HF peak can be found in this band around 0.25–0.3Hz. The power in the HF band is attributed to vagal (parasympathetic)

stimulation of the heart and the HF peak has been shown to correspond to the parasympathetic modulation at a frequency equal to the breathing rate [36], a phenomenon known as respiratory sinus arrhythmia (RSA) [37], [38].

- The low frequency (LF) band, from 0.04 to 0.15 Hz. A characteristic peak can usually be found at 0.1 Hz. Widespread consensus has yet to be reached regarding the physiological correlate for the power in this band [11]. While some authors argue that this band is mainly influenced by sympathetic stimulation of the heart, this has been frequently challenged [39]. Another physiological interpretation for this band is that it reflects the baroreceptor reflex frequency response [36], [39].
- The very low frequency (VLF) band, from 0.0033 to 0.04 Hz. Although this band accounts for a large portion of the spectral power, it has yet to be fully understood and is usually ignored when citing HRV measures [11], even though loss of VLF power has been associated with many causes of mortality, moreso than LF and HF power loss [40]. While some researchers have speculated that this band is related to hormonal factors such as thermoregulation and the renin-angiotensin system [36], recent works reviewed in Shaffer et al. [40] indicate that the VLF power is in fact an intrinsic property of the heart itself.

Nyquist-Shannon sampling theorem

The frequency-based metrics used to measure HRV are the absolute power present in the spectrum in each of the aforementioned frequency bands (measured in ms^2), the ratio of each of these to the total power in the entire range, and the ratio of LF/HF power [11].

It should be noted that as with any type of frequency analysis, certain factors limit our ability to correctly and accurately estimate the power spectrum in the frequency range of interest. The first is the sampling frequency of the time series, which limits the maximal frequency we can correctly resolve. According to the Nyquist-Shannon sampling theorem, a sampling frequency of f_s Hz would allow us to correctly measure the power at frequencies no higher than $\frac{f_s}{2}$ Hz [41]. This frequency is known as the Nyquist rate and sampling at a lower rate would produce distortions in the higher frequencies, a phenomenon known as aliasing [27]. In the case of an RR interval time series, we have an irregularly sampled signal. We could either resample it at a frequency that would allow us to (at a minimum) resolve the end of the HF band or we could use the Lomb method instead. In the latter case, the effective sampling interval is an average which depends on the total number of beats, *N*, in the recording (i.e., for every N beats there are N-1 intervals):

(16)
$$\Delta t_{avg} = \frac{T}{N-1}$$

where *T* is the duration of the recording. By applying the sampling theorem we can see that the number of beats in the recording determines the maximal resolvable frequency:

(17)
$$f_{max} = \frac{\Delta f_{avg}}{2} = \frac{1}{2\Delta t_{avg}} = \frac{N-1}{2T} \ .$$

Usually we know the maximal frequency of interest and the duration of each segment in advance. Therefore we can impose a lower bound on *N*:

(18)
$$N_{min} = 2Tf_{max} + 1.$$

Consequently, while the Lomb method eliminates the need for resampling and thus prevents the artifacts associated with it, the risk of aliasing is higher with this method because the maximal frequency we can resolve without aliasing greatly depends on the average heart rate in the recording. Extra care must therefore be taken to make sure the Nyquist criterion is met in each analyzed segment.

Frequency resolution

Another factor affecting the accuracy of spectral estimation is the frequency resolution, which affects the *minimal* frequency we can resolve. From (10) we can see that in the case of the DFT, the frequency resolution Δf would be:

$$\Delta f = \frac{f_s}{N} = \frac{1}{NT_s} = \frac{1}{T} \,. \tag{19}$$

Thus, the minimum resolvable frequency is inversely proportional to the total duration of the data segment being analyzed.

A parametric spectrum estimate can provide better frequency resolution for short segments because it is not directly dependent on the data segment length [27]. However, for parametric methods, the model order *p* will need to increase with the sampling frequency in order to resolve low frequencies. This has been studied, e.g., in Carvalho et al. [32]. However, other authors have reported that keeping a constant AR order worked better empirically for HRV analysis [42].

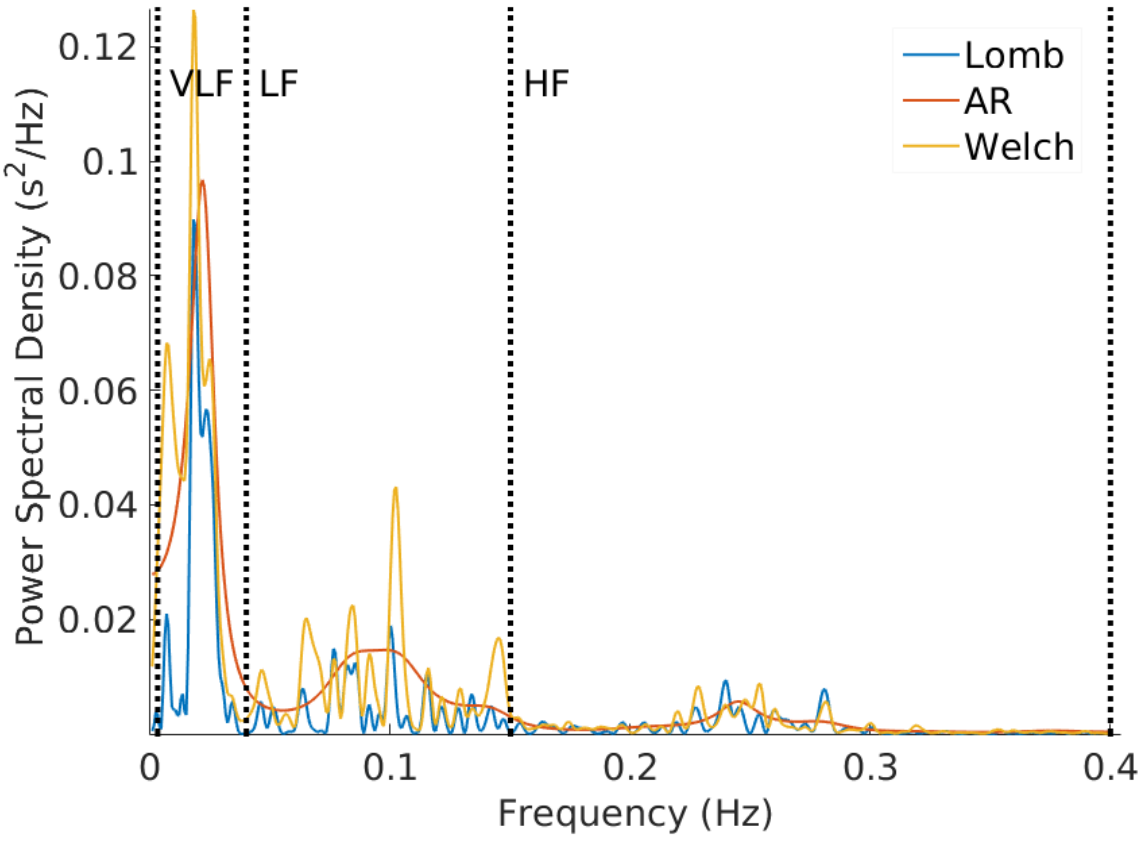

Figure S7: Spectral analysis of fantasia/f1y06. The expected spectral peaks at 0.1 and 0.25 Hz are visible in all three methods; however, the AR method provides a smoother and therefore simpler estimate of the spectrum.

4.3 Nonlinear measures

4.3.1 Poincaré plots

Poincaré plots are a method of visualizing the relationship between consecutive intervals, in which RR_{n+1} is scatter-plotted as a function of RR_n [43], [44]. This makes it a state-space representation of the RR interval signal showing all states (combinations of values) of consecutive intervals that occurred. Usually a Poincaré plot of an RR-interval signal presents as a point-cloud around the line of identity. The most common metrics used to analyze Poincaré plots are *SD1*, the standard deviation of points along the line perpendicular to the line of identity (LOI), and *SD2*, which is the standard deviation of points along the LOI [14]. Visually, *SD1* is a measure of the width of the point cloud while *SD2* is a measure of its length. An ellipse is usually fitted to the points, allowing for visual qualitative inspection and also for ectopic beat detection [44] (intervals outside the ellipse are suspected to be ectopic). It has been previously shown that *SD1* and *SD2* are measures of short- and long-term variability in the RR intervals (respectively) [14], and also that the shape of the plot is related to the spectral power in the low and high frequency ranges [45]. See Figure S8 for an example Poincaré plot.

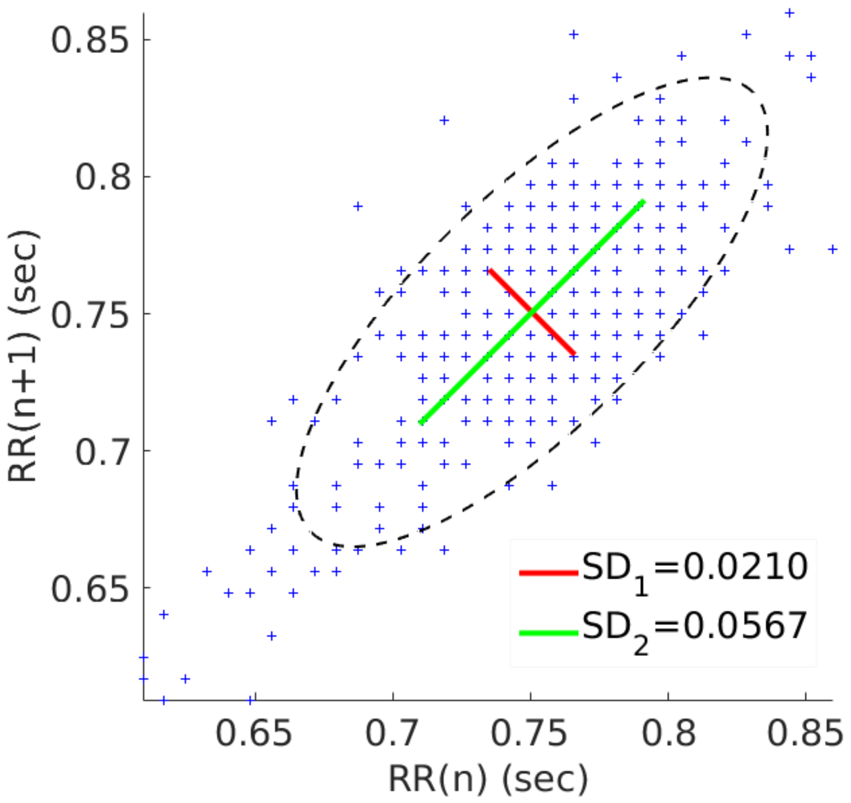

Figure S8: Poincaré plot of a 5-minute segment from record 17453 of the MIT-NSR database. The plot gives a visual representation of the standard metrics SD_1 and SD_2. An ellipse was fitted to two standard deviations using these metrics, in order to show intervals suspected to be ectopic.

4.3.2 Fractal methods

A major drawback of common time and frequency-domain methods is that they assume stationarity of the data (i.e., that the statistical properties of the data do not change with time). However, this is rarely the case with cardiac RR intervals which are known to be comprised of seemingly erratic and chaotic fluctuations including (and especially) in the case of healthy individuals [46]–[48]. The RR interval time series in healthy subjects exhibits self-similar characteristics. The term "self-similar" is usually used to describe fractal phenomena and indeed, the RR interval time series exhibits fractal characteristics which break down under conditions of disease and aging [49]. This breakdown can manifest either with more order (signal becomes less chaotic, more predictable) or with less order (signal becomes more chaotic, behaves like uncorrelated noise) [20], [50]–[53].

Fractal objects

The two main properties of fractal objects are that they are composed of multiple sub-units resembling the whole (self-similarity) and that they have a non-integer (fractional) dimension. Fractal objects are highly related to power-laws. A famous example of this was given by B.B. Mandelbrot regarding the measurement of the length of coastlines [54]. A coastline is usually measured in multiples of unit 'ruler' length G : $L(G) = N \cdot G$ where G is selected to be small enough so that no meaningful features are shorter. Surprisingly, it was empirically discovered that the shorter the ruler length, the longer the coast and, moreover, a power-law relationship emerges when plotting the measured length of the coast *L(G)* as a function of the ruler-length: $L(G) = f \cdot G^{1-d}, d \geq 1$. When this relationship is plotted on a double-log scale, the trendline is linear, with a slope of *1-d*. The constant d is called the 'fractal dimension' of the object. The features of the coastlines, like those of many other shapes in the natural world, have an irregularity to them that can be difficult to quantify. Mandelbrot showed that these objects can be measured and categorized in terms of their fractal dimension. An interesting and important property of power laws is scale invariance. Consider the function $f(x) = a \cdot x^{-k}$. When scaling the argument by a constant c, the value of the function is also scaled proportionally to that constant: $f(c \cdot x) = a \cdot (cx)^{-k} = c^{-k} a \cdot x^{-k} = c^{-k} \cdot f(x)$. This means that power laws of different scales are all equivalent up to constant factors. This scale-invariance is what gives fractal objects their self-similar characteristics [55].

In case of a time dependent process, self-similarity means that the process retains its statistical properties when rescaling to different time scales: statistical scale-invariance [48]. Mathematically, a process *y(t)* is said to be self-similar with scaling exponent α if it has a probability distribution identical to itself after rescaling by a factor of r on the x-axis and of r^{α} on the y-axis:

$$y(t) \equiv r^{\alpha} y(\frac{t}{r}) \quad (20)$$

where $\equiv$ denotes that the probability distribution of both sides is identical. Note that contrary to the spatial case, here we need two different scaling factors because the x- and y-axes correspond to different physical variables. For example, if $r > 1$, we can think of this scaling as zooming-in: the x-axis is magnified by r while the y-axis is magnified by r^{α}.

Detrended Fluctuation Analysis

The parameter α is one possible measure of the complexity of the time series, similar to the concept of the fractal dimension. The challenge is to estimate it from a time series. One method for estimating α directly from time-series data is called Detrended Fluctuation Analysis (DFA) [19]. This method has been previously used on NN interval times series while showing great promise for classification of pathological data [20]. In practice, when working with real-world signals, there is obviously an upper and lower bound to the scale factor *r* (because the signals are bounded), and our ability to estimate the statistical properties accurately is limited. To overcome the first problem, real-world data is first integrated to obtain an

unbounded signal. To overcome the second, the integrated signal is then broken down into windows of n samples, and the statistical properties of the signal are estimated separately in each window using some metric and then averaged. Denote this metric as the average fluctuation, *F(n)*. Given a signal *y[k]* of length *N*, the DFA algorithm defines *F(n)* as the root mean square difference between each sample *y[k]*, and the linear trend in that sample box $y_n[k]$:

(21) $$F(n) = \sqrt{\frac{1}{N}\sum_{k=0}^{N-1}(y[k] - y_n[k])^2}\ .$$

Figure S9 shows the boxing process and the local trend that is subtracted in each box. The process of splitting the signal into boxes of length n and calculating *F(n)* is repeated for all possible values of *n* (1 to N). The outcome of this process is that the x-axis scale parameter r is mapped to the size of the windows through which we view the signal, and the y-axis scale parameter r^{α} is mapped to the average fluctuation at that window size. Plotting *F(n)* as a function of *n* can therefore be thought of as plotting r^{α} vs. r a power-law graph. The self-similarity parameter, α, is therefore estimated by linear regression of the slope on a log-log plot of *F(n)* vs. *n* [48]. See the example in Figure S10.

Interestingly, the value of α estimated from a signal can be used to classify its autocorrelation function and also its power spectrum [48]. For a white-noise process, in which each sample is completely uncorrelated with any other, α will have a value of 0.5. Thus, processes with alpha very close to 0.5 behave as random uncorrelated noise—we can expect their autocorrelation function to be approximately zero for non-zero lags and their power-spectral density to be approximately flat. Signals with $0.5 < \alpha \leq 1$ have an autocorrelation function which obeys power-law behavior: $R(\tau) \propto \tau^{-y}$. Because the power spectrum is given by taking the Fourier transform of the autocorrelation, the spectrum also exhibits power-law scaling and, in fact, $S(f) \propto \tau f^{\beta}$ where $\beta = 2\alpha - 1$. Two additional important cases are $\alpha = \beta = 1$, corresponding to $1/f$ or pink noise, often a characteristic of physiological processes, and $\alpha = 3/2,\ \beta = 2$, which corresponds to Brownian noise, the integral of white noise. Figure S11 B-C shows the autocorrelation functions and DFA curves of such processes.

Goldberger et. al. [46] have found that the power spectrum of healthy heartbeat intervals obeys a power-law form with a $1/f$ type distribution ($\beta = 1$). This type of power distribution was also previously known to be the most ubiquitous when it comes to physiological and natural data. A $1/f$ distribution can be thought of as middle ground between the uncorrelated randomness of white noise ($\beta = 0$) and the more regular, smoother, features of Brownian noise ($\beta = 2$). However, when considering the complexity or information content in the signal (as opposed to its irregularity), $1/f$ noise was previously shown to be the most complex [56]. See Figure S11 B-D for a visual comparison of complexity and irregularity of these three cases. The β scaling exponent can be estimated from the power-spectral density of a time series. For this paper we estimated it based on a PSD derived from an AR model of order 24, by taking the slope of a line fitted with linear regression to the log-log PSD over the VLF frequency band. The DFA algorithm was implemented based on Peng et al. [19] and the PhysioNet implementation [57]. Note that the major drawback of directly estimating β is that spectral estimates assume stationarity of the input, while the DFA method requires no such assumption.

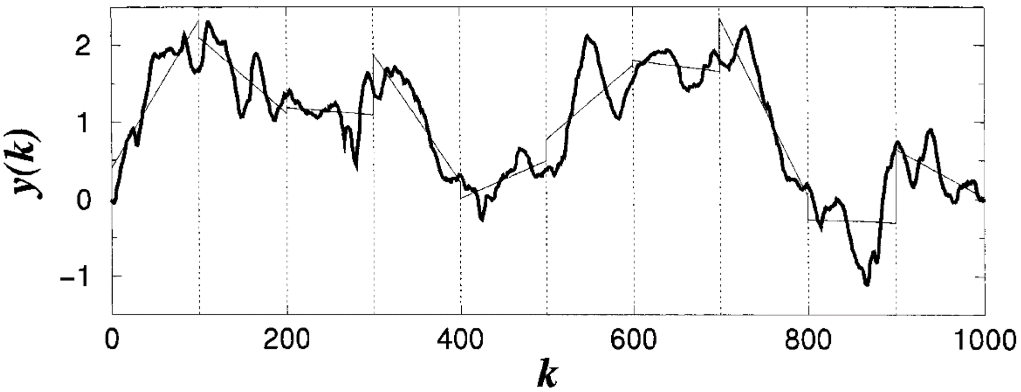

Figure S9: An example signal y[k] broken into 10 boxes of length n=100. The piecewise-continuous line is the box-local linear trend, $y_n[k]$. Adapted from Peng et al [58].

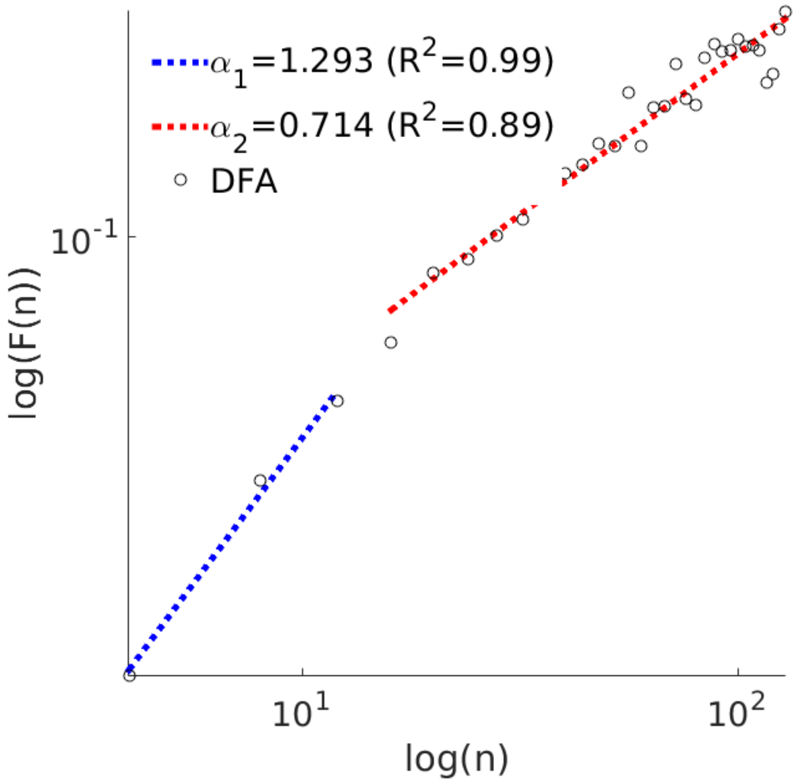

Figure S10: Log-log plot and linear regression for estimation of the DFA slope. 5-minutes of data from record 17453 of the MIT-NSR database was used [16].

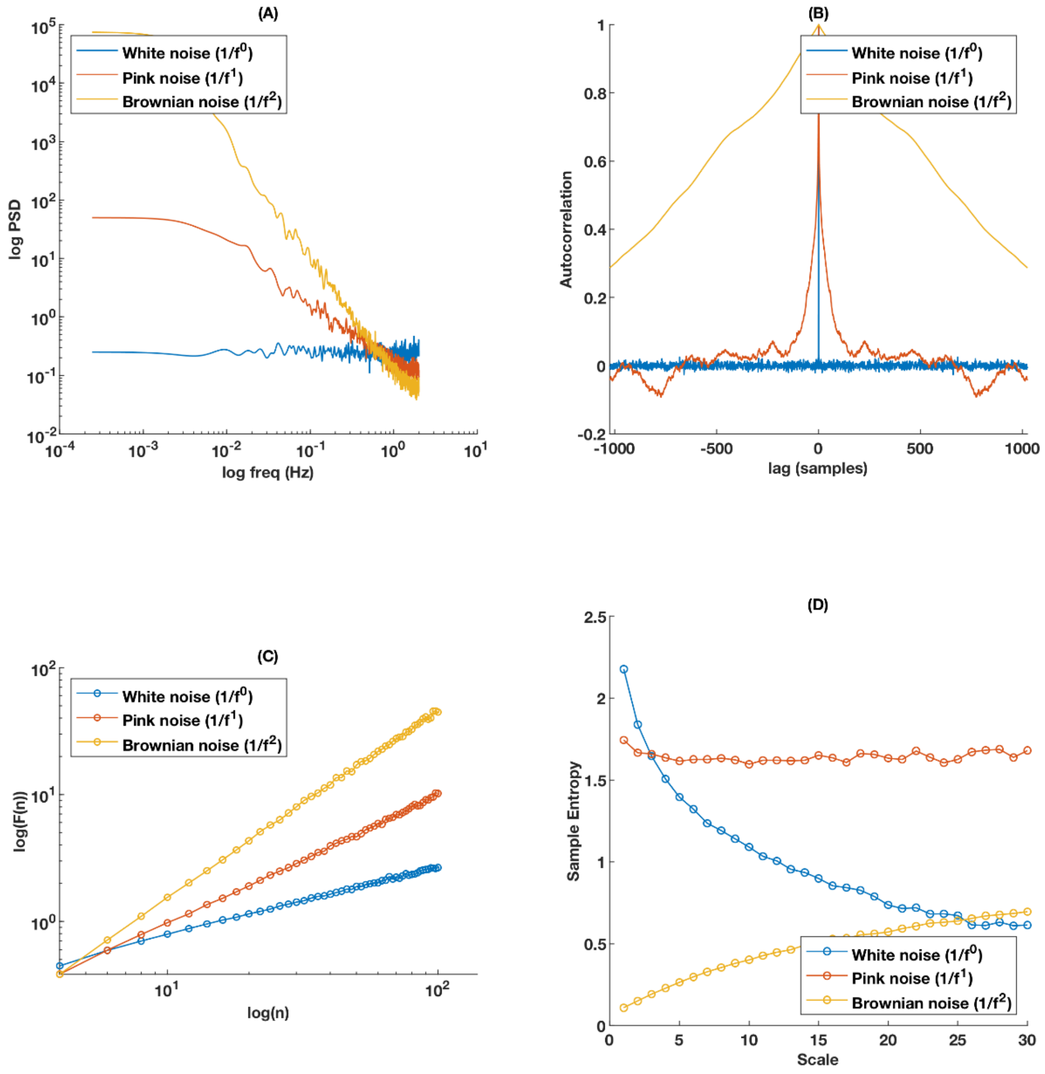

Figure S11: Complexity and irregularity comparison of three stochastic noise benchmarks. **(A)** PSD plots on a double logarithmic scale allowing visual estimation of the power slope β. We can see the white noise process has a relatively flat spectrum (β=0), as expected. The pink and Brownian noise processes have an uneven power distribution, with β=1 and β=2 respectively. **(B)** Autocorrelation function of each process, a visualization of their respective irregularity and complexity. The white noise case exhibits maximal irregularity but without structural features; the Brownian case exhibits predictable (high autocorrelation) and less irregular (smoother) behavior; the pink noise process exhibits both high irregularity and long-range correlations (complex structural features). **(C)** DFA plots, showing a good linear fit (i.e., a power-law scaling exists in the data) and clearly different slopes (corresponding to values of α) in each case. As predicted by the theory, the values of α seen here are 0.5, 1 and 1.5 for white noise, pink noise and Brownian noise respectively. **(D)** MSE curves of the three benchmark processes. Pink noise, a model for physiological processes, exhibits high MSE values at all scales. Brownian noise, which is significantly more predictable, exhibits low MSE, and the white noise case shows high values only at the lowest scales.

4.3.3 Entropy-based methods

The entropy of a stochastic signal, first defined by Shannon [41], is a measure of the average amount of information it contains. For a discrete random variable *X* with a probability measure *P(X)*, the entropy is defined as

(22) $$H(X) = \mathbb{E}\left[-\log\left(P(X)\right)\right] = -\sum_{i=1}^{N} P(X = x_i)\log(P(X = x_i))$$

where $x_i, i = 1, \dots, N$ are the *N* discrete values that *X* can take. A stochastic process is a sequence of random variables $X_i, \dots, X_n$ sampled at sequential points in time. In the case of digital signals, $X_i$are discrete and all take on the same set of possible values (e.g. 0–255). Generally, the value of each sample depends on all previous samples, and therefore a joint probability $P(X_i, \dots, X_n)$ is used. As shown in Cover and Thomas [59], the entropy of a stochastic process with *n* time samples is therefore:

(23) $$H_n = H(X_i, \dots, X_n) = \mathbb{E}[-\log(P(X_i, \dots, X_n))] =$$
$$= -\sum_{i_1=1}^{N} \dots \sum_{i_n=1}^{N} P\left(X_1 = x_{i_1}, \dots, X_n = x_{i_n}\right) \log\left(P\left(\left(X_1 = x_{i_1}, \dots, X_n = x_{i_n}\right)\right)\right).$$

Entropy increases with the amount of irregularity in a signal; predictable signals (e.g., a periodic waveform) have very low entropy, while completely random signals such as white noise have high entropy. This stems directly from the definition—events with a low probability will produce higher values of entropy. It can be shown that the maximal possible entropy occurs for a uniformly distributed random variable and, in the case of a stochastic process, for an uncorrelated random process taking on uniformly distributed values at each point in time [59].

Pathology of the heart usually reduces the fractal dynamics present in the heart rate, therefore diminishing the complex long-range correlations found in the healthy heart rate [49]. There is a relationship between a signal's complexity, influenced by pathology, and its irregularity (measured by entropy). Thus, various methods based on the concept of entropy have been proposed for studying the complexity of biological signals [60]. A prominent entropy-based complexity measure is Sample Entropy [61], which measures the irregularity of a signal by looking at the probability that two similar sequences of length m in the signal are also similar for length *m+1*. More formally, given a time series $u[n], n = 1, \dots, N$, define a sequence of length-*m* vectors starting at each sample,

(24) $$u_m(i) = \{u[i], u[i + 1], \dots, u[i + m]\},\ i = 1,2, \dots, N - m$$

and denote

(25) $$n_i^m(r) = |\{d(u_m(i), u_m(j)) \le r : i \ne j, 1 \le i, j \ne N - m\},$$

which is the number of vectors within distance *r* of $u_m(i)$, with respect to the distance measure:

(26) $$d(u, v) = \max_i |u[i] - v[i]|\ ,$$

which is the l_∞-norm. These vectors are denoted as *template matches* of length *m*. Note that *i* only goes up to *N-m,* so templates of length *,m+1* can also be defined at each start index *i*, and that the requirement $i \ne j$ for $n_i^m(r)$ prevents counting a template as a match for itself. The *r* parameter is usually chosen as a small percentage of the signal's standard deviation. The probability of a match existing for a template starting at index *i* can be estimated by

(27) $$p_i^m(r) = \frac{1}{N-m-1} n_i^m(r)$$

because there are $N-m-1$ total possible matches when excluding self matches ($i \neq j$). The total probability that two sequences of length m in the signal will match is therefore

(28) $$p^m(r) = \frac{1}{N-m}\sum_{i=1}^{N-m} p_i^m(r)$$

since there are $N-m$ template start points. Given the above definitions, the Sample Entropy of $u[t]$ is defined as

(29) $$SampEn(m,r) = -\ln\frac{p^{m+1}(r)}{p^m(r)}$$

$$= -\frac{\ln\sum_{i=1}^{N-m} n_i^{m+1}(r)}{\sum_{i=1}^{N-m} n_i^m(r)}.$$

The Sample Entropy measure is therefore an estimate of the conditional probability that a sequence in the signal of length $m+1$ will match a template, given that the first m samples match [61]; see Figure S12. Note that as with any entropy measure, Sample Entropy is maximal for uncorrelated random noise, because in such a case m matching samples do not provide any information regarding the sample $m+1$. Thus, the ratio of probabilities in equation (28) will be small. Inversely, for a highly predictable and correlated signal (e.g. slow oscillations) the Sample Entropy will be low.

The main drawback of entropy-based methods is that they measure irregularity as a proxy for complexity. However, irregularity is not equivalent to complexity. Complexity in this context depends on the degree of information content in the signal, or the meaningful structure it contains [62]. While complex signals can be highly irregular, irregular signals are not necessarily complex (e.g. uncorrelated noise). This presents a major limitation for using entropy methods as a complexity measure of physiological data. Indeed, as discussed previously, pathology and aging states cause a loss of fractal complexity, which can manifest in either less random or more random interval time series and thus increase or decrease the regularity of the signal. A possible reason for this limitation is that these measures analyze only the shortest time scale in the signal (two adjacent samples), while the complexity of physiological signals comes from their fractal dynamics, which produce long-range correlations on multiple time scales. To quantify the complexity of physiological data, we ideally need a measure which will be low for both cases of complexity loss and take multiple time scales into account.

Multiscale Entropy (MSE) [63] is a complexity measure based on Sample Entropy that aims to overcome the aforesaid limitations of Sample Entropy as a complexity measure for physiological data. To calculate the MSE of a signal $x[n]$ of length N, we first define a "coarse-grained" signal $x^\tau[n]$, where $\tau = 1, \ldots, M$ represents a time scale, and $M \ll N$:

(30) $$x^\tau[n] = \frac{1}{\tau}\sum_{i=(n-1)\tau+1}^{n\tau} x[i],\ 1 \leq n \leq \frac{N}{\tau}.$$

This signal is simply the average of τ adjacent samples in non-overlapping windows of the original signal. After generating $x^\tau[n]$ for multiple scales, the Sample Entropy is calculated for each of them and plotted as a function of the scale τ, producing the MSE curve. This curve is examined to determine the complexity of a signal instead of generating just a single value as the complexity measure. Note that $\tau = 1$ gives us $x^\tau[n] = x[n]$, and therefore the MSE value at the first scale is equal to the Sample Entropy of $x[n]$. Figure S11 shows MSE curves for white, pink and Brownian noise processes. We can see that in contrast to using Sample Entropy at a single scale, MSE is a promising measure of physiological complexity that indeed produces lower values in the cases corresponding to loss of physiological complexity. As we can see Figure S11, MSE curves can be a useful tool for identification of pathology states. Using an MSE curve as a complexity measure, we can say that a signal is considered more complex than another if its entropy is

higher for the majority of time scales. A monotonic decrease in entropy with scale indicates that a signal only contains information on the smallest scale [63].

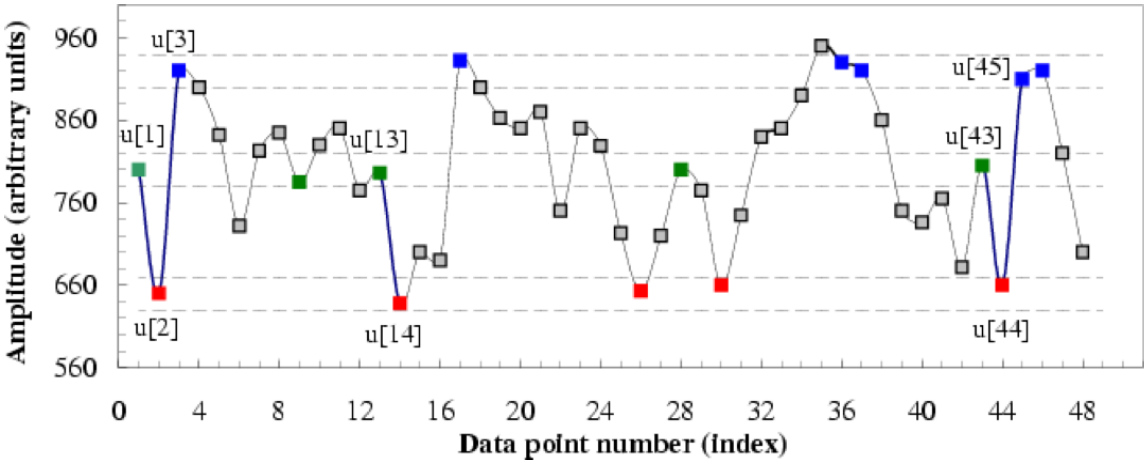

Figure S12: An example of template matching performed by the Sample Entropy algorithm for m=2. The first m+1 samples are used as a template and the dashed lines represent an error interval of width r around each of the template samples. We can see that a partial match of length m exists at samples 13–14, and a complete match of length m+1 exists at samples 43–45. The matching process is performed for each possible template in the signal, and the total number of m-length and m+1-length matches are counted. Adapted from Costa et al. [50].

References

[1] I. Silva *et al.*, "Robust detection of heart beats in multimodal data," *Physio Meas*, vol. 36, no. 8, pp. 1629–44, Aug. 2015.

[2] M. Llamedo and J. Martínez, "QRS detectors performance comparison in public databases," *Comput Cardiol*, vol. 41, pp. 357–60, 2014.

[3] A. E. W. Johnson, J. Behar, F. Andreotti, G. D. Clifford, and J. Oster, "Multimodal heart beat detection using signal quality indices," *Physiol Meas*, vol. 36, no. 8, p. 1665, 2015.

[4] G. Hanton and Y. Rabemampianina, "The electrocardiogram of the Beagle dog: reference values and effect of sex, genetic strain, body position and heart rate," *Lab Anim*, vol. 40, no. 2, pp. 123–36, Apr. 2006.

[5] P. P. Cintra *et al.*, "Cardiorespiratory and electrocardiographic effects of methadone or morphine in the perioperative period in anesthetized dogs with continuous rate infusion of propofol and submitted to ovariohysterectomy," *Semin. Ciências Agrárias*, vol. 38, no. 1, pp. 209–20, 2017.

[6] B. Lord, A. Boswood, and A. Petrie, "Electrocardiography of the normal domestic pet rabbit.," *Vet Rec*, vol. 167, no. 25, pp. 961–5, 2010.

[7] J. Chapel, C. Castillo, J. Hernández, M. Cipone, and J. Benedito, "Electrocardiographic reference values for healthy Netherland Dwarf rabbits and the influence of body position, age and gender," *World Rabbit Sci.*, vol. 25, no. 4, pp. 399–406, 2017.

[8] P. Sysa-Shah, L. L. Sørensen, M. R. Abraham, and K. L. Gabrielson, "Electrocardiographic Characterization of Cardiac Hypertrophy in Mice that Overexpress the ErbB2 Receptor Tyrosine Kinase," *Comp. Med*, vol. 65, no. 4, pp. 295–307, 2015.

[9] G. F. Mitchell, A. Jeron, and G. Koren, "Measurement of heart rate and Q-T interval in the conscious mouse," *Am. J. Physiol. Circ. Physiol.*, vol. 274, no. 3, pp. H747–H751, 1998.

[10] University of Wisconsin-Madison, "Animal Health: Normative Data," *Research Animal Resources Center. University of Wisconsin-Madison*, 2018. [Online]. Available: https://www.rarc.wisc.edu/animal_health/normative_data.html.

[11] M. Malik *et al.*, "Heart rate variability: Standards of measurement, physiological interpretation, and clinical use," *Eur. Hear. J.*, vol. 17, pp. 354–381, 1996.

[12] G. Clifford, "Signal processing methods for heart rate variability," *Dr. Diss. Univ. Oxford*, 2002.

[13] G. Moody, "Spectral analysis of heart rate without resampling," *Comput Cardio*, vol. 20, pp. 715–718, 1993.

[14] M. Brennan, M. Palaniswami, and P. Kamen, "Do existing measures of Poincare plot geometry reflect nonlinear features of heart rate variability?," *IEEE Trans. Biomed. Eng.*, vol. 48, no. 11, pp. 1342–1347, 2001.

[15] J. Piskorski and P. Guzik, "Filtering Poincaré plots," *Comput Meth Sci Tech*, vol. 11, no. 1, pp. 39–48, 2005.

[16] A. L. Goldberger *et al.*, "PhysioBank, PhysioToolkit, and PhysioNet," *Circulation.*, vol. 101, no. 23, 2000.

[17] J. Mietus and A. Goldberger, "Heart Rate Variability Analysis with the HRV Toolkit - PhysioNet," *Available from https//physionet.org/tutorials/hrv-toolkit/*, 2017.

[18] N. Iyengar, C. K. Peng, R. Morin, A. L. Goldberger, and L. A. Lipsitz, "Age-related alterations in the fractal scaling of cardiac interbeat interval dynamics," *Am. J. Physiol. Integr. Comp. Physiol.*, vol. 271, no. 4, pp. R1078–R1084, Oct. 1996.

[19] C.-K. Peng, S. V. Buldyrev, S. Havlin, M. Simons, H. E. Stanley, and A. L. Goldberger, "Mosaic organization of DNA nucleotides," *Phys. Rev. E*, vol. 49, no. 2, pp. 1685–1689, Feb. 1994.

[20] C. - K. Peng, S. Havlin, H. E. Stanley, and A. L. Goldberger, "Quantification of scaling exponents and crossover phenomena in nonstationary heartbeat time series," *Chaos An Interdiscip. J. Nonlinear Sci.*, vol. 5, no. 1, pp. 82–87, Mar. 1995.

[21] K. Schmidt-Nielsen, *Scaling: why is animal size so important?* Cambridge University Press, 1984.

[22] W. R. Stahl, "Scaling of respiratory variables in mammals," *J. Appl. Physiol.*, vol. 22, no. 3, pp. 453–460, Mar. 1967.

[23] D. Ewing, J. Neilson, and P. Travis, "New method for assessing cardiac parasympathetic activity using 24 hour electrocardiograms," *Heart*, vol. 52, no. 4, pp. 396–402, 1984.

[24] J. Bigger, R. E. K. Thomas, J. L. Fleiss, L. M. Rolnitzky, R. C. Steinman, and J. P. Miller., "Components of heart rate variability measured during healing of acute myocardial infarction," *Am. J. Cardiol.*, vol. 61, no. 4, pp. 208–215, 1998.

[25] J. E. Mietus, C. K. Peng, I. Henry, R. L. Goldsmith, and A. L. Goldberger, "The pNNx files: re-examining a widely used heart rate variability measure," *Heart*, vol. 88, no. 4, 2002.

[26] M. D. Costa, R. B. Davis, and A. L. Goldberger, "Heart Rate Fragmentation: A New Approach to the Analysis of Cardiac Interbeat Interval Dynamics.," *Front Physiol*, vol. 8, p. 255, 2017.

[27] B. Porat, *A Course in Digital Signal Processing*. John Wiley, 1996.

[28] S. Orfanidis, *Optimum signal processing: an introduction*, Macmillan. 1988.

[29] P. Welch, "The use of fast Fourier transform for the estimation of power spectra: A method based on time averaging over short, modified periodograms," *IEEE T Acoust Speech*, vol. 15, no. 2, pp. 70–73, Jun. 1967.

[30] G. Walker, "On Periodicity in Series of Related Terms," *Proc. R. Soc. A Math. Phys. Eng. Sci.*, vol. 131, no. 818, pp. 518–532, Jun. 1931.

[31] G. Udny Yule, "On a Method of Investigating Periodicities in Disturbed Series, with Special Reference to Wolfer's Sunspot Numbers," *Philos. Trans. R. Soc. London Ser. A*, vol. 226, pp. 267–298, 1927.

[32] J. L. H. Carvalho, A. F. Rocha, I. dos Santos, C. Itiki, L. F. Junqueira, and F. A. O. Nascimento, "Study on the optimal order for the auto-regressive time-frequency analysis of heart rate variability," *Eng. Med. Biol. Soc. 2003. Proc. 25th Annu. Int. Conf. IEEE*, vol. 3, pp. 2621–2624, 2003.

[33] L. Wasserman, "Probability Redux: Stochastic Processes," Springer Science & Business Media, 2004, pp. 381–401.

[34] N. R. Lomb, "Least-squares frequency analysis of unequally spaced data," *Astrophys Sp. Sci*, vol. 39, no. 2, pp. 447–462, Feb. 1976.

[35] J. D. Scargle, "Studies in astronomical time series analysis. II-Statistical aspects of spectral analysis of unevenly spaced data," *Astrophys. J.*, vol. 263, pp. 835–53, 1982.

[36] S. Akselrod, D. Gordon, F. A. Ubel, D. C. Shannon, A. C. Berger, and R. J. Cohen, “Power spectrum analysis of heart rate fluctuation: a quantitative probe of beat-to-beat cardiovascular control,” *Science.*, vol. 213, no. 4504, pp. 220–222, 1981.

[37] P. E. McSharry, G. D. Clifford, L. Tarassenko, and L. A. Smith, “A dynamical model for generating synthetic electrocardiogram signals,” *IEEE Trans. Biomed. Eng.*, vol. 50, no. 3, pp. 289–294, Mar. 2003.

[38] G. E. Billman, “Heart rate variability - a historical perspective.,” *Front. Physiol.*, vol. 2, p. 86, 2011.

[39] R. McCraty, M. Atkinson, and D. Tomasino, *Science of the Heart - Exploring the Role of the Heart in Human Performance*. HeartMath Research Center, Institute of HeartMath, 2015.

[40] F. Shaffer, R. McCraty, and C. L. Zerr, “A healthy heart is not a metronome: an integrative review of the heart’s anatomy and heart rate variability.,” *Front. Psychol.*, vol. 5, p. 1040, 2014.

[41] C. E. Shannon, “A mathematical theory of communication,” *Bell Syst. Tech. J.*, vol. 27, no. 3, pp. 379–423, Jan. 1948.

[42] A. Boardman, F. S. Schlindwein, A. P. Rocha, and A. Leite, “A study on the optimum order of autoregressive models for heart rate variability,” *Physiol Meas*, vol. 23, no. 2, pp. 325–336, May 2002.

[43] J. Piskorski and P. Guzik, “Geometry of the Poincaré plot of RR intervals and its asymmetry in healthy adults,” *Physiol. Meas.*, vol. 28, no. 3, pp. 287–300, Mar. 2007.

[44] A. C. C. Yang, “Poincare Plots: A Mini-Review,” *Physionet*, 2006. [Online]. Available: https://www.physionet.org/events/hrv-2006/yang.pdf.

[45] M. Brennan, M. Palaniswami, and P. Kamen, “Poincare plot interpretation using a physiological model of HRV based on a network of oscillators,” *Am Physiol. Soc*, vol. 283, no. 5, pp. H1873–H1886, 2002.

[46] A. L. Goldberger, V. Bhargava, B. J. West, and A. J. Mandell, “On a mechanism of cardiac electrical stability. The fractal hypothesis,” *Biophys. J.*, vol. 48, no. 3, pp. 525–528, Sep. 1985.

[47] A. L. Goldberger, D. R. Rigney, and B. J. West, “Chaos and fractals in human physiology,” *Sci. Am.*, vol. 262, no. 2, pp. 42–49, 1990.

[48] C. K. Peng, J. M. Hausdorff, A. L. Goldberger, and J. Walleczek, “Fractal mechanisms in neuronal control: human heartbeat and gait dynamics in health and disease,” in *Self-organized biological dynamics and nonlinear control: toward understanding complexity, chaos and emergent function in living systems. Cambridge University Press*, 2006, pp. 66–96.

[49] A. L. Goldberger, L. A. N. Amaral, J. M. Hausdorff, P. C. Ivanov, C.-K. Peng, and H. E. Stanley, “Fractal dynamics in physiology: alterations with disease and aging.,” *Proc. Natl. Acad. Sci. U. S. A.*, vol. 99 Suppl 1, no. suppl 1, pp. 2466–72, Feb. 2002.

[50] M. Costa, A. L. Goldberger, and C.-K. Peng, “Multiscale entropy analysis of biological signals,” *Phys. Rev. E*, vol. 71, no. 2, p. 021906, Feb. 2005.

[51] S. Guzzetti *et al.*, “Non-linear dynamics and chaotic indices in heart rate variability of normal subjects and heart-transplanted patients,” *Cardiovasc. Res.*, vol. 31, no. 3, pp. 441–446, Mar. 1996.

[52] L. Bergfeldt and Y. Haga, “Power spectral and Poincaré plot characteristics in sinus node dysfunction,” *J. Appl. Physiol.*, vol. 94, no. 6, pp. 2217–2224, Jun. 2003.

[53] P. C. Ivanov *et al.*, “Scaling and universality in heart rate variability distributions,” *Phys. A Stat. Mech. its Appl.*, vol. 249, no. 1–4, pp. 587–593, Jan. 1998.

[54] B. Mandelbrot, “How long is the coast of Britain? Statistical self-similarity and fractional dimension.,” *Science*, vol. 156, no. 3775, pp. 636–8, May 1967.

[55] B. Mandelbrot and R. Pignoni, *The fractal geometry of nature*, WH freeman. 1983.

[56] Y.-C. Zhang, “Complexity and 1/f noise. A phase space approach,” *J. Phys. I*, vol. 1, no. 7, pp. 971–977, Jul. 1991.

[57] A. L. Goldberger *et al.*, “PhysioBank, PhysioToolkit, and PhysioNet: components of a new research resource for complex physiologic signals.,” *Circulation*, vol. 101, no. 23, pp. E215-20, Jun. 2000.

[58] C. K. Peng, S. Havlin, J. M. Hausdorff, J. E. Mietus, H. E. Stanley, and A. L. Goldberger, “Fractal mechanisms and heart rate dynamics: long-range correlations and their breakdown with disease,” *J. Electrocardiol.*, vol. 28, pp. 59–65, 1995.

[59] T. M. Cover and J. A. Thomas, *Elements of information theory*, John Wiley. 2012.

[60] S. M. Pincus, “Assessing Serial Irregularity and Its Implications for Health,” *Ann. N. Y. Acad. Sci.*, vol. 954, no. 1, pp. 245–267, Jan. 2001.

[61] J. S. Richman and J. R. Moorman, “Physiological time-series analysis using approximate entropy and sample entropy,” *Am J Physiol-Heart C*, vol. 278, no. 6, pp. H2039–H2049, Jun. 2000.

[62] M. D. Costa, C.-K. Peng, and A. L. Goldberger, “Multiscale Analysis of Heart Rate Dynamics: Entropy and Time Irreversibility Measures,” *Cardiovasc. Eng.*, vol. 8, no. 2, pp. 88–93, Jun. 2008.

[63] M. Costa, A. L. Goldberger, and C.-K. Peng, “Multiscale Entropy Analysis of Complex Physiologic Time Series,” *Phys. Rev. Lett.*, vol. 89, no. 6, p. 068102, Jul. 2002.

5. Additional Tables

Table S4: Dog database with reference annotations

File name	Length (hr:min:sec)	Total number of annotations	Percentage of bad quality annotations
**Dog_01**	00:04:29	514	0%
**Dog_02**	00:06:31	862	15.9%
**Dog_03**	00:06:34	762	6%
**Dog_04**	00:04:52	586	8.8%
**Dog_05**	00:05:54	636	0%
**Dog_06**	00:05:35	752	0%
**Dog_07**	00:04:37	456	0%
**Dog_08**	00:05:50	849	0%
**Dog_09**	00:06:01	808	0%
**Dog_10**	00:05:54	652	0%
**Dog_11**	00:05:49	585	0%
**Dog_12**	00:04:09	513	0%
**Dog_13**	00:04:35	414	6.5%
**Dog_14**	00:06:48	731	0%
**Dog_15**	00:05:33	689	0%
**Dog_16**	00:04:44	361	0%
**Dog_17**	00:06:01	701	6%
**Total**	01:33:55	10,871	2.7%

Table S5: Rabbit database with reference annotations

File name	Length (hr:min:sec)	Total number of annotations	Percentage of bad quality annotations
**Rabbit_01_part_1**	00:15:41	3,304	0.2%
**Rabbit_01_part_2**	00:04:59	1,065	0%
**Rabbit_01_part_3**	00:04:53	1,048	0%
**Rabbit_01_part_4**	00:07:45	1,747	0%
**Rabbit_01_part_5**	00:12:22	2,725	0%
**Rabbit_02_part_1**	00:05:39	1,196	1.5%
**Rabbit_02_part_2**	00:08:23	1,771	0.2%
**Rabbit_02_part_3**	00:09:04	1,935	0%
**Rabbit_02_part_4**	00:26:00	5,433	2.8%
**Rabbit_02_part_5**	00:13:12	2,852	2.2%
**Rabbit_03_part_1**	00:05:16	1,336	0%
**Rabbit_03_part_2**	00:07:46	2,086	0%
**Rabbit_03_part_3**	00:06:52	1,864	0.1%
**Rabbit_03_part_4**	00:05:59	1,626	0%
**Rabbit_03_part_5**	00:14:54	4,046	1.4%
**Rabbit_03_part_6**	00:13:48	3,775	0%
**Rabbit_03_part_7**	00:08:21	2,256	3.5%
**Rabbit_04_part_1**	00:18:27	4,791	1.7%
**Rabbit_04_part_2**	00:15:23	3,927	0%
**Rabbit_04_part_3**	00:06:28	1,669	1.6%
**Total**	03:31:13	50,452	1.0%

Table S6: Mouse database with reference annotations

File name	Length (hr:min:sec)	Total number of annotations	Percentage of bad quality annotations
**Mouse_1**	00:19:20	8,467	0%
**Mouse_2**	00:13:53	7,721	0%
**Mouse_3**	00:29:20	16,674	0.6%
**Mouse_4**	00:33:18	17,147	0.1%
**Mouse_5**	00:29:38	17,923	0.1%
**Mouse_6**	00:20:40	9,656	0%
**Mouse_7**	00:22:20	12,183	2.4%
**Mouse_8**	00:39:38	20,094	0%
**Total**	03:28:07	109,865	0.4%

Supplement 2: Procedure for HVR benchmark

The details of the procedure for benchmarking between the HRV toolbox from PhysioZoo and PhysioNet are provided:

Comparison Scheme

1. Create two results tables, one for PhysioZoo and one for PhysioNet. The columns in each table correspond to HRV measures and its rows to different analysis windows from the datasets.
2. For each record and each analysis window:
    1. Load official PhysioNet annotations for that window.
    2. Calculate RR intervals based on the annotations.
    3. Sanity check: skip window if RR interval distribution satisfy one of the following criteria:
        - $\mu_{RR} + \sigma_{RR} \geq RR_{\max}$, OR
        - $\mu_{RR} - \sigma_{RR} \leq RR_{\min}$
    4. Apply pre-filtering to remove suspected non-NN intervals:
        1. Range based filter, then
        2. Moving average filter
    5. The filter results are the NN intervals to be used throughout. Write them to a file so they can be used as input for the PhysioNet tools.
    6. Calculate the following metrics with PhysioZoo
        1. NN/RR ratio
        2. Time domain: AVNN, SDNN, RMSSD, pNN50
        3. Frequency domain: Total power, VLF/LF/HF band power, LF to HF ratio
        4. Nonlinear: MSE, DFA
    7. Calculate the same metrics with PhysioNet's tools using the NN intervals input file:
        1. Run the PhysioNet get_hrv script from the HRV Toolkit to calculate the time and frequency-domain metrics.
        2. Run the PhysioNet MSE program.
        3. Run the PhysioNet DFA program.
    8. Collect the results into a new row in each result table.
3. Create a comparison table: each row is an HRV metric and each column is a comparison metric.
    1. Calculate the RMSE for each HRV metric (column) in the results tables.
    2. Calculate the normalized RMSE (NRMSE) by dividing each RMSE by the mean of the PhysioNet results column.

Parameter		Description	Value	Units
**Pre-filtering**				
	$RR_{\min}$	Min physiological RR interval	0.4	seconds
	$RR_{\max}$	Max physiological RR interval	2.0	seconds
	L_w	Length of moving-average window (each side)	20	seconds
	T_w	Moving-average threshold ratio of sample value and window value	0.2	n.u.
**Frequency**				
	PSD method	Method for PSD calculation	Welch; Lomb	
	Window function	Window function applied to signal before calculating PSD	Hamming; Rectwin	
**MSE**				
	$S_{\max}$	Maximal scale MSE is computed up to	20	n.u.
	r	Template matching threshold	0.2	n.u.
	m	Template base length	2	n.u.
	normalize	Whether to normalize input to zero mean and unit variance	True	
**DFA**				
	$n_{\min}$	Minimal scale DFA is computed at	4	n.u.
	$n_{\max}$	Maximal scale DFA is computed at	64	n.u.

Table S7: Parameters for comparison of the HRV toolboxes.
